# Combining Electrochemiluminescence Detection with Aptamer‐Gated Indicator Releasing Mesoporous Nanoparticles Enables ppt Sensitivity for Strip‐Based Rapid Tests

**DOI:** 10.1002/anie.202110744

**Published:** 2021-11-09

**Authors:** Estela Climent, Knut Rurack

**Affiliations:** ^1^ Chemical and Optical Sensing Division Bundesanstalt für Materialforschung und -prüfung (BAM) Richard-Willstätter-Str. 11 12489 Berlin Germany

**Keywords:** electrochemiluminiscence, hybrid materials, penicillin, signal amplification, test strip analysis

## Abstract

The combination of electrogenerated chemiluminescence (ECL) and aptamer‐gated indicator delivering (gAID) magnetic mesoporous silica nanoparticles embedded into glass fibre paper functionalised with poly(ethyleneglycol) and *N*‐(3‐triethoxysilylpropyl)diethanolamine allowed the development of a rapid test that detects penicillin directly in diluted milk down to 50±9 ppt in <5 min. Covalent attachment of the aptamer “cap” to the silica scaffold enabled pore closure through non‐covalent electrostatic interactions with surface amino groups, while binding of penicillin led to a folding‐up of the aptamer thus releasing the ECL reporter Ru(bpy)_3_^2+^ previously loaded into the material and letting it be detected after lateral flow by a smartphone camera upon electrochemical excitation with a screen printed electrode inserted into a 3D‐printed holder. The approach is simple, generic and presents advantages with respect to sensitivity, measurement uncertainty and robustness compared with conventional fluorescence or electrochemical detection, especially for point‐of‐need analyses of challenging matrices and analytes at ultra‐trace levels.

## Graphical abstract

The best of both worlds in one strip, i.e., electrochemical excitation and luminescence emission, together with aptamer‐gated hybrid nanomaterials enables the detection of penicillin directly in milk at ultra‐trace levels in less than 5 min with a simple test strip and a mobile communication device.

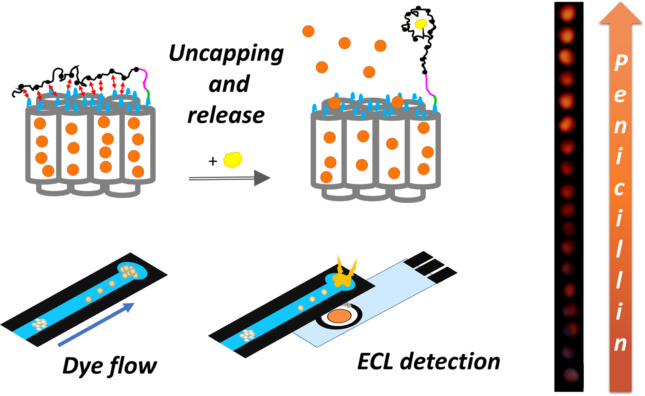

## Introduction

Fuelled by their key role in helping to contain the SARS‐CoV‐2 pandemic especially through the fast identification of contagious yet potentially asymptomatic individuals, rapid tests have perhaps received unprecedented attention in societies around the globe during the last year.^[1]^ However, besides their current prominence in medical diagnostics, rapid tests and assays have also become increasingly important in other areas such as food,^[2]^ agriculture and forestry,^[3]^ security and forensics^[4]^ or the environment^[5]^ in the last decade. Their advantage is that they can be used outside of a laboratory by untrained personnel directly at a point of need, minimizing time between first suspicion and first decision taking. In this regard, paper‐based sensors are an attractive and emerging class of devices^[6]^ because they fulfil the prerequisites of the World Health Organization's ASSURED principle: they are affordable, sensitive, specific, user‐friendly, rapid and robust, equipment free and deliverable to end‐users.^[7]^ The physical, chemical and mechanical properties of cellulose or glass fibre paper in combination with the simplicity of preparation render these materials very interesting also in terms of resource‐effective alternatives for device production technologies.^[8]^ Furthermore, the ubiquity of mobile communication devices with powerful computing capabilities and onboard cameras have led to a situation in which a large majority of the global population in principle has a powerful detector in hand that is especially suited for taking photographs of flat substrates such as paper strips and analysing their content. While the use of smartphones as detectors has started a decade ago,^[9]^ only the advent of affordable 3D printing technologies and OTG (on‐the‐go) electronics have boosted the field,^[10]^ making the fabrication of cases to fit on a phone simple and affordable and the adaption to new phone models with different size or camera position straightforward.^[11]^ OTG electronics allow for facile and autonomous integration of microelectrodes and light‐emitting diodes (LEDs) for electrochemical and fluorescence measurements into such holders,^[12]^ leaving much more room for assay development than the photographing of coloured areas.

Because of these advantages, many efforts have been undertaken to design sensory nanomaterials for paper‐based point‐of‐care diagnostics.^[13]^ Better robustness, sensitivity and multiplexing capabilities have been achieved by tuning the properties of the membranes in combination with the use of mobile phones for data analysis,^[14]^ improving the performance of such devices.^[15]^ Most of the current paper‐based rapid tests rely on colorimetric,^[16]^ fluorescence^[17]^ or electrochemical detection.^[18]^ However, many of these assays show weaknesses in terms of specificity, sensitivity, accuracy and precision or the capability for multiplexed detection.^[19]^ Whereas specificity is connected to the recognition (bio)chemistry, colorimetric detection, which is still the prevalent method in rapid tests, is almost exclusively relying on gold nanoparticles (AuNPs) which are decorated with biomacromolecular binders, mainly antibodies. Although AuNPs possess distinctly higher molar absorption coefficients than organic dyes or coloured inorganic ions, such assays are limited with respect to sensitivity, especially when used with visual (naked eye) inspection. In addition, such colorimetric tests are also primarily employed for biomolecular analytes as the immunoassay formats commonly employed, sandwich and competitive, often show inferior sensitivity in small‐molecule analysis.^[20]^ Furthermore, multiplexing of AuNP‐based colorimetric assays requires spatial separation and cannot rely on an identification via different colours. Fluorescence detection in contrast can be measured straightforwardly with a smartphone camera and allows for fluorescence colour multiplexing but has drawbacks in signal‐to‐noise ratio especially when used with paper supports, scattering light significantly. Electrochemical detection on the other hand would require additional accessory and multianalyte detection is a challenge. Thus, a promising approach is the combination of both techniques in electrogenerated chemiluminescence or electrochemiluminescence (ECL) detection.^[21]^ ECL dispenses with a light source for excitation, thus reducing noise significantly, and, when compared with the widely used colorimetric assays, possesses all the advantages of fluorescence.[^21^, ^22^] Although ECL detection for paper‐based rapid tests has been realized a decade ago,^[22]^ up to now only considerably few examples have been reported, most of them for the detection of heavy metal ions, DNA or protein biomarkers as well as whole cells.^[23]^ However, many of these reports only show the applicability of ECL sensing on paper and many of the examples are characterized by rather high detection limits.

In ECL, a luminescence signal is generated by a chemical reaction that is initiated and controlled by the application of an electrical potential. Since signal generation is only taking place at the electrode and only for the duration of an applied potential, ECL is a highly localized and controlled detection method. The paramount advantage of ECL compared with fluorescence is that it does not require a light source for excitation, allowing to reach exceptionally high signal‐to‐noise ratios. This is important in two aspects, i.e., for non‐transparent and scattering supports such as paper and for turbid sample media such as milk, wastewater or body fluids, when these need to be analysed without clean‐up. As only comparatively few compounds show ECL emission under ambient conditions in aqueous media, tris(2,2′‐bipyridine)ruthenium(II) chloride (Ru(bpy)_3_^2+^) is the most frequently used ECL reporter.^[24]^ Furthermore, powerful ECL sensing is only possible when Ru(bpy)_3_^2+^ is combined with a co‐reactant, such as a secondary or tertiary amine,^[25]^ leading to a desired enhancement of the ECL signal and allowing for the detection in aqueous solution under a constant potential. Another practical advantage of Ru(bpy)_3_^2+^‐based ECL is the wavelength range of emission (600–750 nm) that can be easily detected by smartphone cameras.^[26]^

Based on our experience in developing powerful yet simple test strip‐based sensing systems for point‐of‐need scenarios,^[27]^ a challenge remains the ultra‐trace detection of analytes in complex media, e.g., of pollutants such as pesticides or antibiotics in foodstuff such as milk. Consider a milk truck driver who can use a test during every stop at a farm when collecting fresh milk on the daily tour, to screen for the presence of antibiotics such as penicillin before accepting the milk to be filled into the tank, thus avoiding potential contamination of the entire load. The economic and health benefits are immediately obvious. Such a test should be simple to use for an untrained person, provide a reliable result in short time, record the result automatically for documentation purposes and should be very sensitive as, for instance, the maximum residue level (MRL) for penicillin in milk is as low as 4 μg kg^−1^.^[28]^ To develop such a test, we combined the advantages of ECL detection with our highly sensitive, selective and modular nanoparticle‐based signalling approach that utilizes gated indicator release^[27b]^ and implemented them on a lateral flow‐type test strip with smartphone readout. As we have recently demonstrated, such an approach can be expanded facilely into a test that remains simple yet allows to analyse a small number of analytes at the same time.^[29]^ Such low‐number multiplexing would be highly desirable also for our milk truck driver as it would allow the testing for various lead contaminants at every farm in a time‐saving and straightforward way. We report here for the first time how the synergistic use of ECL detection and gated indicator release in rapid paper‐based assays allows to determine antibiotics such as penicillin in a challenging matrix like milk down to the ppt level in less than 5 min of overall assay time.

## Results and Discussion

**Scheme 1:**
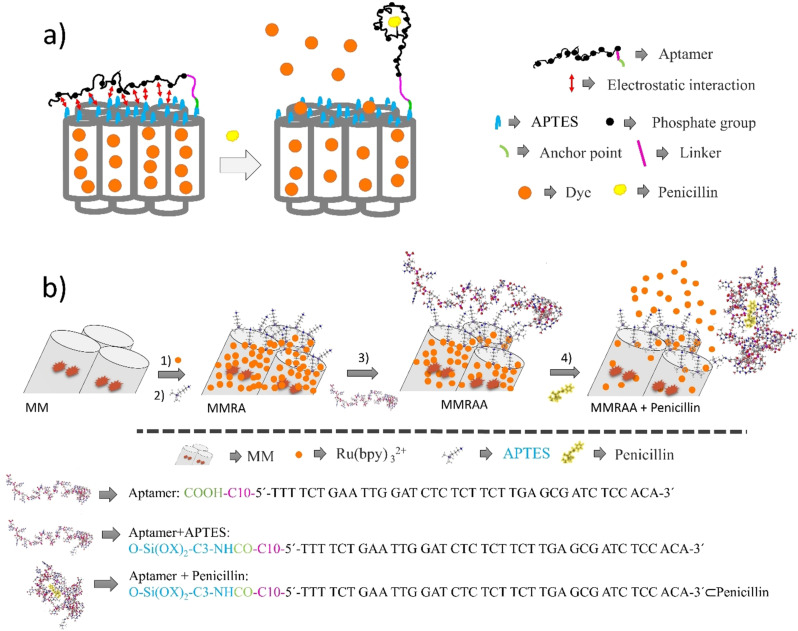
(a) Gating mechanism of the gAID system as described in the text. For better understanding, the relevant dimensions are as follows: length of aptamer with 39 nucleotides=ca. 13 nm, pore diameter=2.4 nm (see text), wall thickness=ca. 2.2 nm (see text), length of APTES group=ca. 0.45 nm, diameter of Ru(bpy)_3_^2+^=ca. 1.2 nm, ratio of surface coverage of aptamer to APTES=ca. 1:1000 times (Table S1). (b) Preparation sequence (1–3) and mode of operation (3,4) of the gAID system. 1) Loading of Ru(bpy)_3_^2+^ into the mesopores. 2) Functionalisation of the outer surface with APTES moieties. 3) Covalent grafting of an aptamer moiety through its 5′‐terminus appended 1‐carboxy decyl linker onto the surface via EDC/NHS coupling chemistry. 4) Upon advent of penicillin, the aptamer folds up and the analyte is bound in the designated binding pocket, entailing release of the dyes. As each aptamer is bound to the particle surface through only one covalent bond but multiple non‐covalent interactions, whenever an analyte molecule approaches and binds to its designated binding motif, the aptamer folds up, the binding pocket closes, and the aptamer is locked in a conformation where minimal electrostatic interactions with surface APTES groups can occur. The pore is open.

An aptamer‐gated indicator delivery (gAID) system was chosen as chemical recognition element because aptamers, which are DNA sequences with high selectivity and affinity for target proteins,^[30]^ small molecules^[31]^ or metal ions,^[32]^ are an attractive alternative to antibody‐gated systems while showing superior versatility and modularity.^[33]^ The principle of gated indicator delivery signalling is as follows (Scheme S1).^[34]^ A porous scaffold material, commonly mesoporous silica nanoparticles, is loaded with indicator molecules and coated with (bio)chemical entities as so‐called gatekeepers, which are grafted covalently to the scaffold's outer surface. Bulky entities such as biomacromolecules are then bound to these gatekeepers, usually in a non‐covalent fashion, capping the pores and blocking release of the indicator cargo (Scheme S1a). The system is designed in such a way that a target analyte binds stronger to either gatekeeper or cap than the two gating partners bind to each other, leading to a dissociation of this pore closing ensemble and hence allowing for a release of the cargo. The result is a chemical signal amplification as few analyte molecules react with the gating ensemble and lead to the delivery of many more reporter molecules, diffusing from the pores into the surrounding solution.^[35]^ Especially in combination with biochemical gating, such systems have shown already good sensitivity and selectivity in simple assay formats. With respect to gAID systems, a few have been already reported in the literature.^[36]^ However, in those cases, the aptamer as such has been non‐covalently coated onto the surface of the porous host material and is fully displaced from the inorganic scaffold after binding of the corresponding analyte, the binding event entailing a refolding of the aptamer that facilitates desorption from the surface (Scheme S1a). This approach is inconvenient when other components of a real sample can lead to unintended desorption and thus false‐positive release of indicator. When aiming at the detection of antibiotics in milk, such a scenario is rather likely because of the electrolyte content and the presence of various surface‐active compounds in milk. Instead of antibody‐based gAID systems with which we have worked so far,[^27b^, ^29^] aptamers seemed more attractive to us here because they offer better possibilities of defined covalent chemical attachment to the outer surface of the porous host, including adjustment of linker length between anchor point and binding region. Our principle is thus different (Scheme S1b, Scheme 1): in the closed state, the aptamer is in a loose, open, unfolded form and is capping the pores by electrostatic interaction with an excess of functional groups on the surface, like for single‐strand DNA‐gated systems.^[37]^ In essence, each aptamer is covalently attached to the particle surface via its 5′ terminus through one anchor point (green fragment, Scheme 1 a) while the negatively charged phosphate groups on the oligonucleotide backbone (black dots) can non‐covalently interact (red arrows) with positively charged ammonium groups of the excess amino silane moieties of the primary functional surface coating (blue fragments). Use of a propyl‐amido‐decyl linker (magenta fragment) allows the aptamer strand to bend over and orient horizontally to the surface so that electrostatic interactions can occur (red arrows). While the covalent bond fixes the aptamer, the multiple non‐covalent interactions are dynamic, the many aptamers on one particle thus forming a constantly changing monolayer‐type network of nucleotide strands on the particle surface. If an analyte molecule (yellow shape) comes close to the binding region and can dock to the motif, a conformational rearrangement of the aptamer strand takes place that leads to a folding up of the aptamer in the complex, away from the particle surface, inhibiting the non‐covalent interactions and opening the pores for the dye (orange dots) to diffuse out. The covalent attachment of the aptamer thus ensures that, instead of lifting the entire gate from it hinges, analyte binding simply induces that the gate swings open.

Specifically, the newly developed material comprises a magnetic mesoporous silica nanoparticle (MSN) scaffold that is loaded with Ru(bpy)_3_^2+^ and contains a penicillin‐selective aptamer^[38]^ covalently attached to the outer surface, ensuring residence of the cargo in the pores in the absence of an analyte (Scheme 1 b). Upon binding of penicillin, the aptamer changes its conformation, folds up at the distal end of the surface thus opening the pores and leading to the release of a large number of Ru(bpy)_3_^2+^ reporters. The detailed synthesis, functionalisation and characterisation of the materials are given in Sections 2–4, Figures S1–S5, Tables S1, S2 of the Supporting Information.

Before testing the performance of the material, the ECL signalling of Ru(bpy)_3_^2+^ in solution was optimised for sensitivity by screening several co‐reactants and SPE electrodes (see Sections 5.1–5.3, Figures S6–S8, Table S3 in Supporting Information). As a result, we employed an SPE electrode containing gold as a working electrode, platin as a counter‐electrode and silver as a reference electrode (AT250), in conjunction with *N*‐butyldiethanolamine (NBEA) as a co‐reactant at a concentration of 5 mM.

The sensing material **MMRAA** was prepared by suspending magnetic mesoporous nanoparticles **MM** of MCM‐41‐type in a highly concentrated Ru(bpy)_3_^2+^ solution in acetonitrile to load the maximum amount of dye into the calcinated scaffold, yielding **MMR** (see Section 3 in Supporting Information). Mesoporous particles with a magnetic core were chosen because they endow the system with more flexibility in particle handling. Next, amino groups were covalently attached to the outer surface by condensation of 3‐aminopropyltriethoxysilane (APTES), resulting in **MMRA**, before the penicillin‐selective aptamer, COOH‐C10‐5′‐TTT TCT GAA TTG GAT CTC TCT TCT TGA GCG ATC TCC ACA‐3′,^[38]^ was grafted covalently through its terminal carboxylic acid group to the amino groups of **MMRA** via an active‐ester‐method (see Section 3 in Supporting Information). The presence of the mesoporous structure of **MM** was confirmed with nitrogen adsorption‐desorption isotherms, small and wide‐angle X‐ray scattering (SAXS/WAXS), scanning electron microscopy (SEM) and transmission electron microscopy (TEM) analysis. SEM and TEM images revealed that the as‐prepared nanoparticles **MM** were spherical with radii between 70–110 nm, i.e., with an average size of 205±34 nm, encapsulating iron oxide nanoparticles (**IO‐NPs**) with diameters of 6.5±1.1 nm. Furthermore, the specific surface area (1009 m^2^ g^−1^), pore diameter (2.4 nm) and pore volume (0.67 cm^3^ g^−1^) were determined by porosimetry studies using the Barrett‐Joyner‐Halenda (BJH) and Brunauer‐Emmett‐Teller (BET) models on the adsorption branch of the isotherm for analysis. SAXS measurements provided the lattice cell parameter (4.59±0.06 nm) which, together with the pore size, allowed the wall thickness to be estimated to 2.18 nm (see Section 4 in Supporting Information). The respective contents of APTES and Ru(bpy)_3_^2+^ on and in the material were determined to 1.03 and 0.26 mmol g solid^−1^ for **MMRA** from elemental analysis, thermogravimetry and spectrophotometric measurements, respectively. Successful condensation of the aptamer was revealed by zeta potential and STEM‐EDX measurements. The latter showed an increase in the phosphorus and sulphur content for the material. The aptamer content was estimated from spectrophotometry through a standard addition method by measuring the increase in absorbance at 260 nm, yielding contents of 0.7±0.3 μmol g solid^−1^. This amount corresponds to a coupling efficiency of ca. 70 % of the aptamer added during the synthesis (see Section 4 in Supporting Information). Zeta potential measurements were performed in water and buffers of different pH (water at pH 7; MES 100 mM, pH 5; PB 10 mM, pH 8), all of them showing a negative displacement of the zeta potential after covalent anchoring of the aptamer moieties to the surface of **MMRAA** (displacement of ca. 40 mV in both buffered media; 80 mV in water), due to reduction of the net positive charge of the aminated particles. The latter includes covalent amide bond formation as well as electrostatic interaction between phosphate groups of the aptamer backbone with the excess amino groups in their protonated ammonium state on the surface. After addition of 2 ppm of penicillin, a small displacement to less negative zeta potential values was observed at neutral pH (displacement of ca. +4 mV in PB 10 mM; +15 mV in water), ascribed to the binding with the aptamer producing a conformational rearrangement that leads to a breaking of the non‐covalent interactions and a folding up of the aptamer in the complex, thus de‐shielding amino surface groups during the opening of the pores.

**Figure 1:**
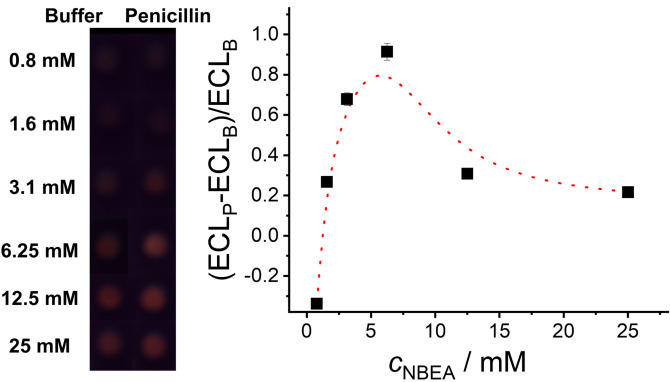
Left: Photographs showing the ECL signal of Ru(bpy)_3_^2+^ released from **MMRAA** into the supernatant in the absence and the presence of 1 ppm of penicillin as a function of the co‐reactant concentration (*c*_NBEA_=0.8–25 mM) in PB (10 mM, pH 8) containing 25 % of milk. Photographs were shot during the application of 1.1 V for 10 s via amperometry measurements. Right: Plot of the corresponding ECL signal as a function of NBEA concentration. The line is only a guide to the eye.

To assess whether the optimized amount of co‐reactant might have to be adjusted for best performance of the gAID system under realistic conditions, **MMRAA** was tested by suspending the material 5 min in the presence and the absence of 1 ppm of penicillin in a mixture of buffer containing different amounts of co‐reactant (0.8–25 mM NBEA) and milk (25 %), keeping in mind that pH control is important because many aptamers retain their integrity and binding behaviour until ca. pH 9^[39]^ and the ECL emission yield of Ru(bpy)_3_^2+^ is highest at pH 9 (Figure S9). Figure 1 shows the ECL emitted in absence and in presence of penicillin as a function of the NBEA concentration. Whereas *c*_NBEA_ <1.6 mM was not able to produce a significant response, the optimum was found again in the range of 3 mM < *c*_NBEA_ <6 mM, which let us use an NBEA concentration of 5 mM in the subsequent studies. It should be noted that the interplay of electrolyte content (buffer concentration) and co‐reactant concentration (the co‐reactant being a base) is critically important for system operation. Too high electrolyte content could interfere with the non‐covalent interaction of aptamer and surface amino groups as well as complex formation^[40]^ and a too high or too low pH would also strengthen or weaken these interactions (p*K*_a_ of APTES=9.6),^[41]^ which would influence blank release or binding kinetics.

Having established the chemical parameters of the assay in suspension, the response kinetics were studied. The response time is an essential feature of every rapid test as it majorly decides about acceptance by the end user. The experiments were carried out as explained before, i.e., in buffered solution (10 mM PB, pH 8; 5 mM NBEA) containing 25 % of cow milk, respectively. Figure S10a shows that the presence of penicillin induced the opening of the pores with the subsequent release of Ru(bpy)_3_^2+^, whereas release was negligibly low in the absence of the analyte, dye release happening on the order of several minutes. This is not extremely fast, but it should be noted that the response kinetics in suspension usually differ from those on strip, the latter often being faster because of active transport in the lateral flow. Since a satisfactory level of signal was reached after 5 min, we proceeded with this timing. The kinetics were also assessed for different amounts of penicillin, at final concentrations between 1 ppb and 10 ppm, showing that the response accelerates with analyte concentration which hints at diffusion control of such assays in suspension (Figure S10b).

**Figure 2:**
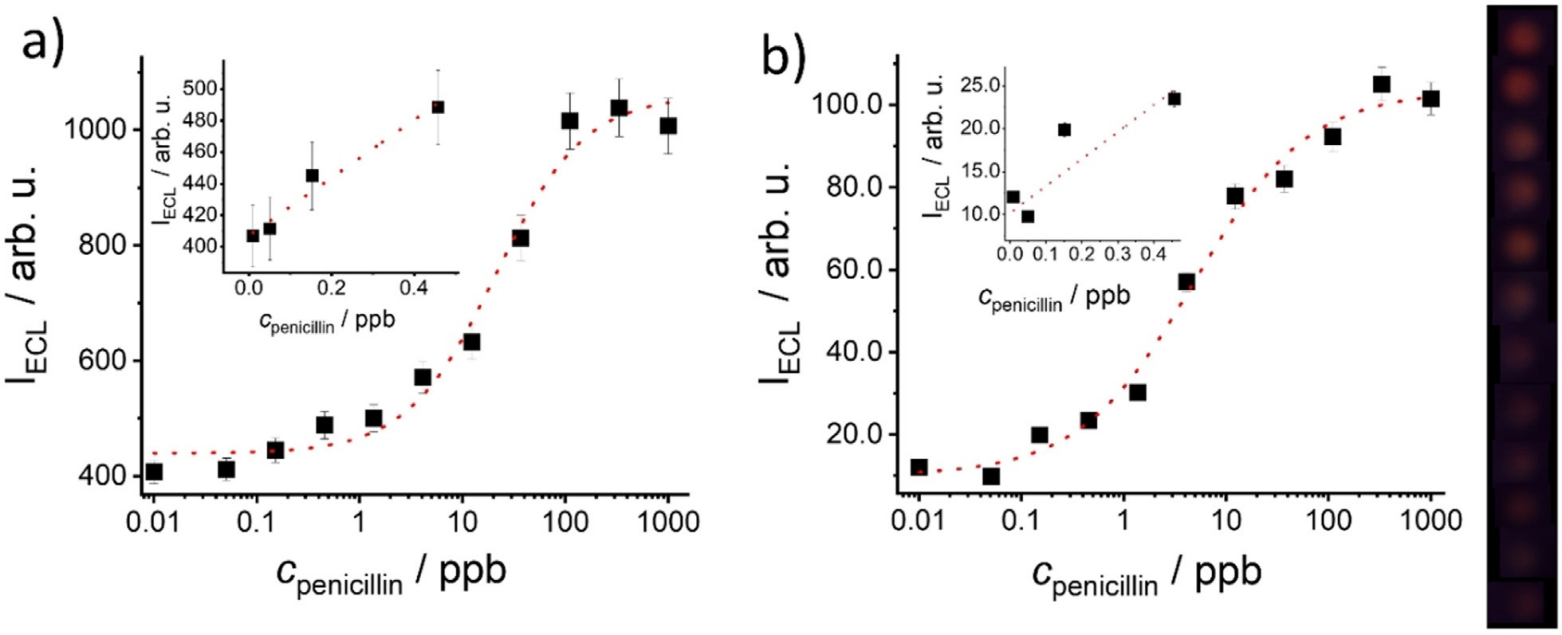
ECL signal of Ru(bpy)_3_^2+^ released from **MMRAA** as a function of penicillin concentration in buffered milk (PB, 10 mM, pH 8; NBEA, 5 mM; 25 % cow milk) after 5 min of reaction measured by a) the spectraECL cell and b) the smartphone setup. The lines exemplify four‐parametric logistic fits. Inset: Zoom into the respective regions of low *c*_penicillin_.

Following a similar procedure, system sensitivity was studied by recoding dye release from **MMRAA** as a function of penicillin concentration after 5 min of reaction by both, ECL (Figure 2) and fluorescence (Figure S11). A correlation between dye release and analyte concentration was observed in all cases, in agreement with conformational changes of the surface‐bound aptamer upon binding of penicillin, opening the pores. However, when the dose‐response curves were fitted to a four‐parametric logistic function,^[42]^ a higher sensitivity was found for ECL compared with fluorescence, yielding limits of detection (LODs) of 0.18±0.07 μg l^−1^ as well as 0.42±0.10 μg l^−1^ for ECL using the smartphone as well as the spectraECL cell and 3.1±0.4 μg l^−1^ when employing the fluorometer (Figure S11). This difference of ca. one order of magnitude is tentatively ascribed to the scattering of milk contained in the medium, which affects fluorescence measurements. When penicillin was present in the lower ppb range (7 ppb), an amplification factor of 120 molecules of Ru(bpy)_3_^2+^ delivered per molecule of penicillin in the sample was estimated. With amounts of 1 ppb of penicillin, an amplification factor of 350 was obtained.

With the aim to reinforce our theory of the sensing mechanism, in which in the closed state of the gAID system the aptamer is in an open form and is capping the pores by interaction with an excess of functional amino groups on the surface, two control materials **MMRAAc** and **MMRCA** were prepared. For **MMRAAc**, a mixture of two different oligonucleotides was grafted to the surface of **MMRA**, the aptamer used for **MMRAA** and a short c‐DNA, COOH‐C10‐5′‐TTT TGT GGA GAT C‐3′, that is partially complementary to the sequence of the penicillin aptamer (Schemes S1c, S2). Using a 1:1 mixture of aptamer and c‐DNA it was expected that the aptamer would (partially) hybridize with the short c‐DNA, inhibiting dye escape through base strand pairing instead of electrostatic interaction with surface amino groups. Kinetic control experiments showed a release of the dye that is virtually identical for **MMRAA** and **MMRAAc** in the absence of analyte, yet that the presence of analyte led to a slower and reduced release for **MMRAAc** in comparison with **MMRAA** (Figure S12a). When **MMRAAc** was treated with different concentrations of penicillin in a similar way as **MMRAA**, a release of dye as a function of *c*_penicillin_ was also observed. However, the sensitivity of **MMRAAc** was ca. one order of magnitude lower than that of **MMRAA** (LOD=24±6 ppb, Figure S13). This reduced sensitivity is tentatively ascribed to an aggravated competition of penicillin for the aptamer once the latter has hybridized to the short c‐DNA strand.

For the second control material **MMRCA**, the aptamer was grafted with an amino group at the 5′ terminus (instead of COOH as in **MMRAA** and **MMRAAc**) to the surface of **MMRC**, which was synthesised by treating **MMRA** with succinic anhydride, converting the surface amino largely into COOH groups (Scheme S2). The aim was to support our consideration of non‐covalent capping by electrostatic attraction between surface ammonium groups and phosphate groups on the aptamer backbone. Expressing a carboxylate‐rich surface it was expected that pore closure would be much less efficient for the aptamer grafted to **MMRC**, i.e., in **MMRCA**. Already during its preparation, distinctly more dye was released when washing **MMRCA** than for **MMRAA** or **MMRAAc**, indicating that the gate was not properly closing the pores. Furthermore, in binding studies a higher dye release compared with **MMRAA** or **MMRAAc** was observed for **MMRCA** in the absence of the analyte and a much lower release in presence of the analyte, indicating that besides pore closure also the gating mechanism was not efficient (Figure S12b). Zeta potential measurements of the control materials supported these observations (Figure S4).Whereas for **MMRAA** and **MMRAAc**, negative displacements of the zeta potential by ca. 75 mV and 85 mV (in H_2_O, pH 7) were observed after covalent anchoring of the aptamer or the aptamer/c‐DNA moieties to the surface of **MMRA**, **MMRCA** showed a displacement of only ca. −40 mV with respect to **MMRC**, presumably because conjugation of one aptamer introduced 39 phosphate groups per one carboxylate anchor group and not because of shielding by non‐covalent interaction. The effect of the analyte was also different, the smallest changes in zeta potential occurring for **MMRCA**, demonstrating that the binding of penicillin by the aptamer produces a conformational rearrangement and pore opening for **MMRAA** and **MMRAAc**, yet not for **MMRCA**.

Having established the sensing behaviour in solution, we moved towards applicability and incorporated the hybrid particles with modified glass fibre strips. Based on previous work in which we have shown that the chemical functionalization of a test strip matrix can significantly improve assay performance,[^27a^, ^43^] we also went through several matrix tailoring cycles by sterically adsorbing or covalently anchoring PEG and *N*‐alkyldiethanolamine moieties on the fibres to arrive at the best material used here. Whereas the PEG moieties preserve the stability of the sensing material **MMRAA** in the paper matrix and facilitate the transport of Ru(bpy)_3_^2+^ due to a reduction of the electrostatic interaction of the positively charged dye with a net negatively charged silanol‐ and silanolate‐expressing surface of the glass fibre paper, the *N*‐alkyldiethanolamine groups enhance the ECL signal of the dye. For that purpose, glass fibre papers (Fusion 5 grade, **GF**) were first modified by adsorption of NBEA and PEG groups on the membranes, yielding **(NP)GF** membranes. Alternatively, *N*‐(3‐triethoxysilylpropyl)diethanolamine (NPEAS) and 3‐[methoxy(polyethyleneoxy)propyl)] trimethoxysilane (PEGS) were grafted covalently to **GF** membranes in toluene, yielding the membrane **NPGF** (see Section 6, Figures S14–S16, Table S4 in Supporting Information).

**Figure 3:**
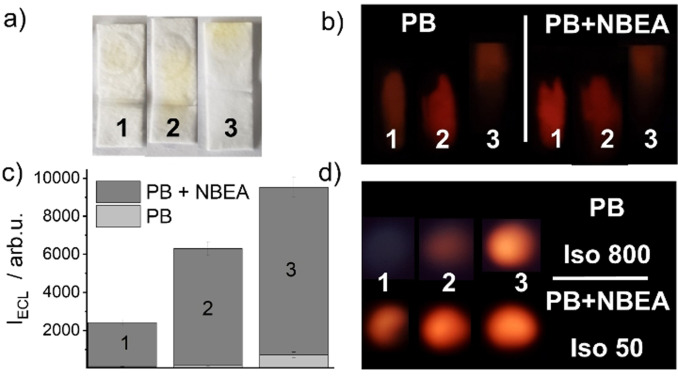
Photographs taken under a) normal daylight and b) LED excitation in a 3D‐printed holder of non‐modified glass fibre strips **GF** (1), **(NP)GF** (2) and **NPGF** (3) membranes. c) ECL signal of Ru(bpy)_3_^2+^ measured for the strips placed on an AT250 SPE electrode at the end of their development using either PB (10 mM, “PB”) or PB (10 mM) and NBEA (25 mM; “PB+NBEA”) as mobile phase with the spectraECL cell; membrane numbering as in a,b). d) ECL images registered with the smartphone setup for the membranes (numbering as in a,b) measured at the end of the developed strips using either PB or PB+NBEA (see a,b) as mobile phase. Note that ISO was reduced ca. 16× when NBEA was present, to avoid ECL signal saturation on the images.

The improvement of the ECL efficiency of the modified papers was assessed by suspending 2.5 μl Ru(bpy)_3_^2+^ solution (1.2 mM) at ca. 7 mm from the bottom and dipping the strips for 2 min into 300 μl of a solution of PB 10 mM (pH 8) or PB 10 mM containing NBEA (25 mM) before recording the ECL as well as the fluorescence emission of released Ru(bpy)_3_^2+^ in zone B (Figure 3). As can be seen in Figure 3 a,b, Ru(bpy)_3_^2+^ was strongly adsorbed on the membranes containing no modification (**GF**) or NBEA and PEG moieties sterically adsorbed (**(NP)GF**). In contrast, when NPEAS and PEGS were covalently grafted to the membranes (**NPGF**), Ru(bpy)_3_^2+^ was much less retained. Interestingly, less fluorescence was observed in both cases for the covalently functionalised strips **NPGF**. Figure 3 c shows the corresponding ECL emission measured for three different strips after development with PB (10 mM) or PB (10 mM) containing NBEA (25 mM). The signal increase from **GF** via **(NP)GF** to **NPGF** is apparent, and a favourably strong reddish orange ECL was especially seen for **NPGF**, which is advantageous with respect to handling and on‐site measurements. Moreover, both, the presence of NBEA and PEG adsorbed in **(NP)GF** as well as the presence of NPEAS and PEGS grafted to **NPGF** were able to enhance the ECL efficiency. In addition, a further strong enhancement was observed in both cases when NBEA was also used in solution (Figure 3 c, examples 2, 3). Thus, membranes **NPGF** containing covalently grafted NPEAS and PEGS moieties were further used.

**Scheme 2:**
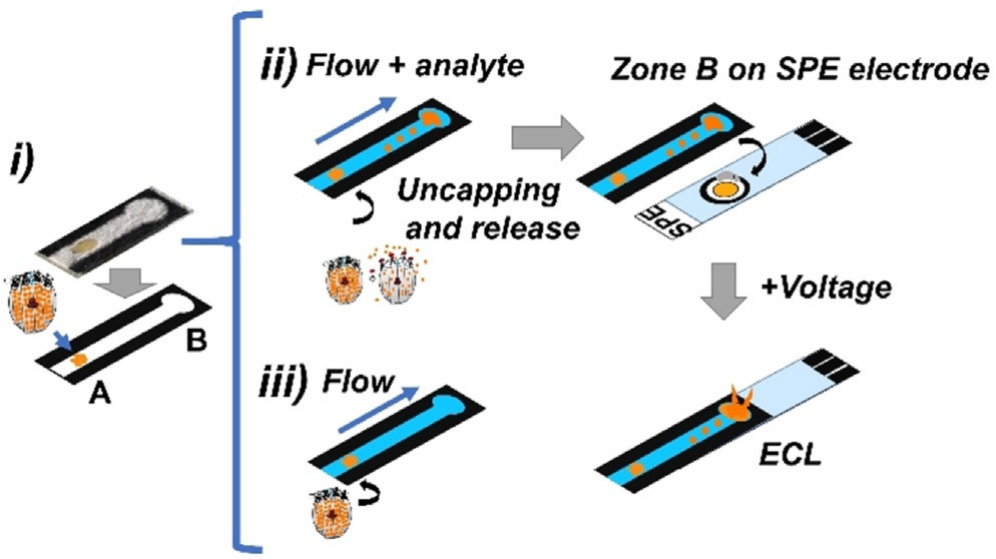
Design and principle of operation of the LFA. i) Composition of the strips containing **MMRAA** in zone A and working principle of dye release from **MMRAA** in ii) the presence and iii) the absence of the analyte. A schematic representation of how zone B of the membrane is placed on the electrode is also shown. In the presence of an analyte, ECL emission is generated at the electrode area upon applying a voltage.

To improve performance through better control of the flow, hydrophobic wax patterns were printed onto **NPGF** membranes by lamination of the printed patterns on aluminium foil. Curing the strips at 110 °C for 1 h led to a melting of the wax into the membrane, imprinting the features across the entire thickness of the paper. Sensing material **MMRAA** was then incorporated into strips **NPGF** by depositing 5 μl of a solution of **MMRAA** in PB (2 mg ml^−1^) at the interaction zone (zone A) of the strip, located ca. 5 mm from one end of the strip (Scheme 2; see Section 6 in Supporting Information). The membranes were analysed with an optical microscope and by SEM, revealing that the incorporation of PEGS and NPEAS groups and the subsequent impregnation with wax led to an expansion of the fibres (Figure S15a). Energy‐dispersive X‐ray spectroscopy (EDX), thermogravimetric (TGA) and elemental analysis (EA) were conducted to qualitatively estimate the amounts of PEGS and NPEAS groups. An increase in mass loss of 6.7 % for **NPGF** compared with neat **GF** membranes was found by TGA (see Section 6 in Supporting Information). Furthermore, this result was in good agreement with EA, which provided amounts of PEGS and NPEAS groups as 0.14±0.02 and 0.09±0.01 mmol g^−1^ glass fibre membrane, corresponding to a total mass loss of 7.1 %.

**Figure 4:**
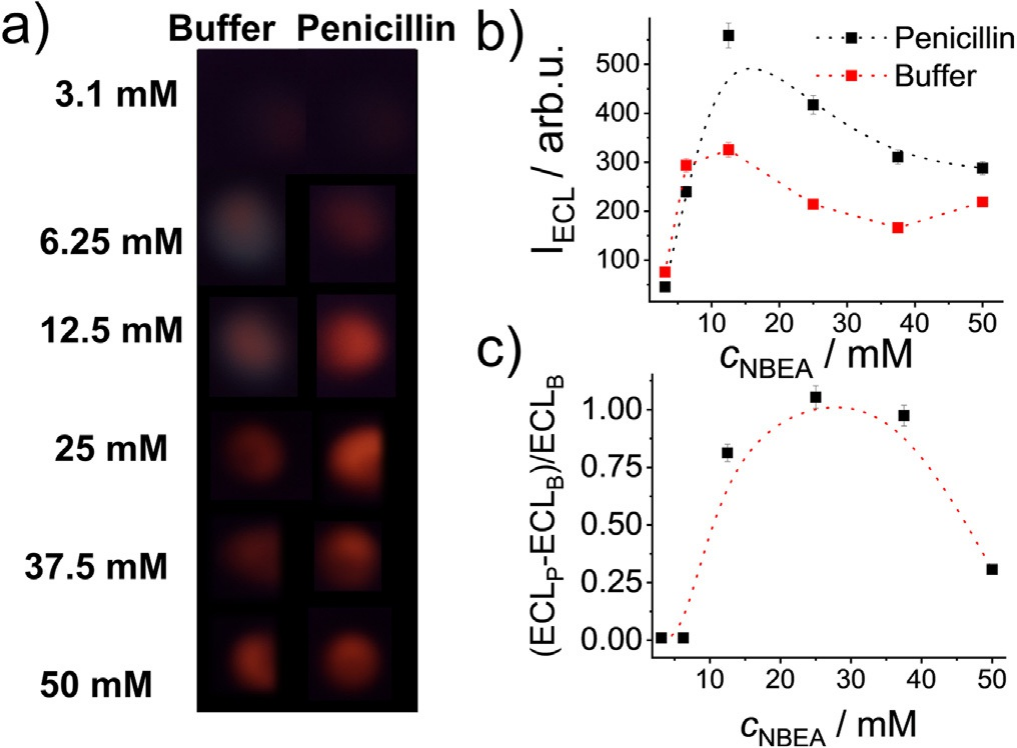
a) Photographs showing the ECL signal of Ru(bpy)_3_^2+^ in the detection zone B in the absence and the presence of 1 ppm of penicillin as a function of co‐reactant concentration (*c*_NBEA_=3.1–50 mM) in the kit solution (PB 10 mM, pH 8) with which the sample (milk) is diluted in a ratio of 3+1 kit solution + sample. Images were registered during application of 1.1 V for 10 s in amperometric mode. b) Plot of the integrated ECL signal as a function of *c*_NBEA_. c) Corresponding plot of the ECL enhancement in presence of 1 ppm of penicillin (ECL_P_) relative to the ECL in absence of penicillin (ECL_B_).

The layout of the sensing membranes was based on designs recently reported by us for gAID‐based lateral flow assays (LFAs),[^27b^, ^29^] containing two different zones, a zone A, in which the sensing material is deposited, and a zone B, in which the released indicators are confined at the distal end of the strip after traveling with the solvent front. Chemical recognition happens in zone A and the ECL signal is measured in zone B (Figure 4 a). When the strip is dipped into a solution that does not contain an analyte, no dye is released, and no signal is detected in zone B because **MMRAA** remains capped. In the presence of the analyte in the sample, a signal proportional to the analyte concentration is detected in zone B because aptamer moieties are rearranged on the surface and dye molecules are released. The much larger **MMRAA** particles remain at the spot of deposition. As a result, the ECL (or the fluorescence) of the reporter molecules released can be quantified with the onboard camera of a smartphone, when the latter is equipped with the respective miniaturized accessories. In addition, the strips were also evaluated with the spectraECL cell, verifying the ECL spectra.

Like for **MMRAA** in suspension, the optimum amount of co‐reactant had to be found for a dipping time of 2 min which yields optimum contrast (Figure S17). Figure 4 shows the corresponding reddish orange ECL light emitted in the absence and the presence of penicillin as a function of the concentration of NBEA. In contrast to the results observed in suspension, a quenching effect was found in the paper experiments at much higher *c*_NBEA_ >25 mM, providing also better signal‐to‐noise ratios when using higher amounts of co‐reactant as in solution (12.5 mM < *c*_NBEA_ <37.5 mM). On strip, lower amounts of NBEA (3–6 mM) induced only a moderate ECL signal; still higher amounts (50 mM) produced a strong release also in absence of the analyte. Following Figure 4, 25 mM of NBEA was chosen for further studies. That the quenching only occurs at higher NBEA concentrations on strip compared to solution is tentatively attributed to the preconcentration of the released dye in zone B and to a partial retention of the co‐reactant in the fibrous matrix itself. To keep the assay simple, NBEA has to be provided together with the buffer in a single sample preparation step, i.e., 1+3 dilution of the sample so that the use of higher amounts is necessary. Handling of another solution and including a pipetting step to apply NBEA directly to zone B was no option for us.

Because of the considerably higher concentration optimum of NBEA for the strip experiments, the influence of pH on the performance of **MMRAA@NPGF** was assessed. 25 mM NBEA in solution were equivalent to pH 9, see above. On the strip with its coated fibres, the microscopic pH can be different, or the strip material can have a certain buffering effect. However, as the pH in the strips is difficult to measure, we repeated the strip assay for dipping solutions with an adjusted pH of 7.0–9.6 in the presence and absence of 250 ppb of penicillin in diluted milk as described before (Figure S18). It was found that the strips showed best and stable performance between pH 8.5–9.0. While the behaviour between pH 7.0–9.0 seems to be primarily dictated by the ECL efficiency of Ru(bpy)_3_^2+^ (see also Figure S9), the high blank release in absence of analyte at pH 9.6 suggests that capping becomes inefficient, because too few surface amino groups are still in their protonated form, the p*K*_a_ of APTES being 9.6.^[41]^ Up to pH 9.0, the binding efficiency of the aptamer thus seems to be largely unaffected by pH.

**Figure 5:**
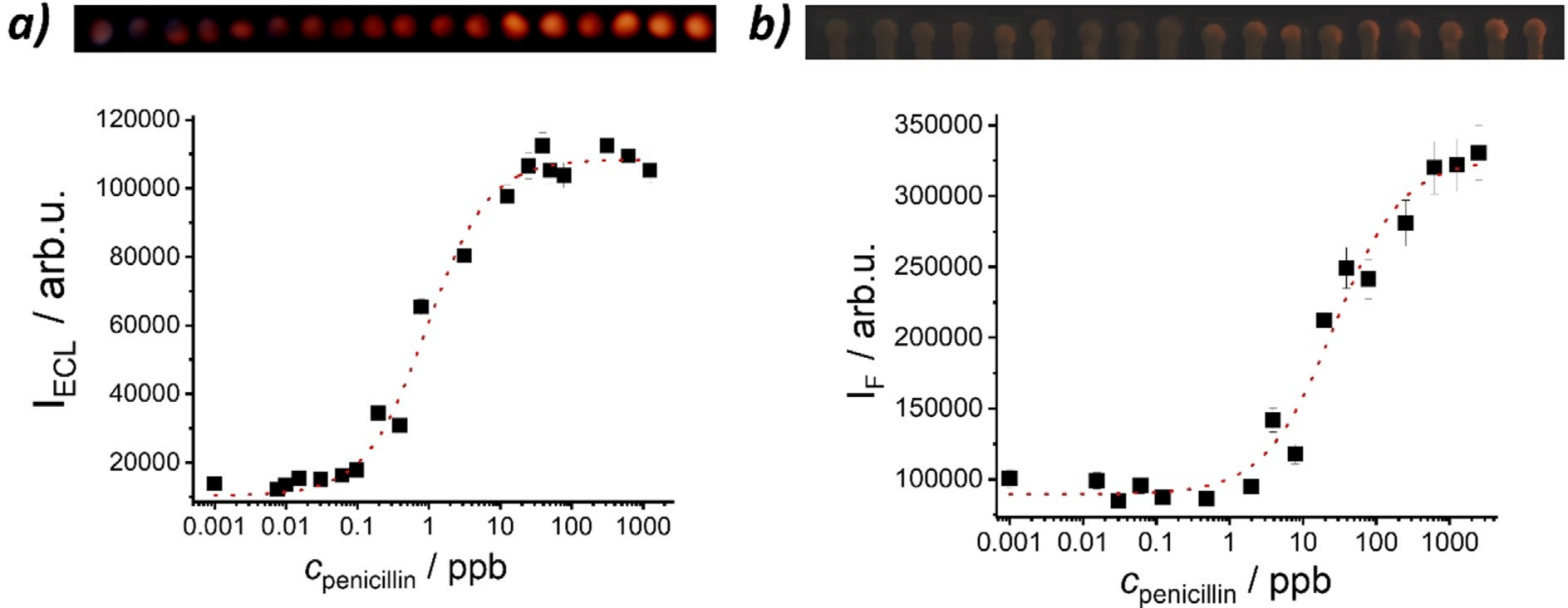
a) Integrated density of light emitted by Ru(bpy)_3_^2+^ released from **MMRAA** as a function of penicillin concentration in milk diluted 1+3 with buffer (PB, 10 mM, pH 8; NBEA, 25 mM) on **NPGF** strips after 2 min of reaction measured with the smartphone and a) ECL or b) fluorescence readout. The lines exemplify four‐parametric logistic fits. Top: Corresponding images analysed for data acquisition (increasing analyte concentration from left to right). For b) the brightness was increased by 15 % from the original images for better visualization.

The sensitivity of **MMRAA@NPGF** was evaluated next, dipping them into 300 μl of buffered solutions (PB, 10 mM, pH 8; NBEA, 25 mM) containing 25 % of milk and different amounts of penicillin as described before. As can be seen in Figure 5, in both cases, ECL and fluorescence detection, an increase of dye release was observed as a function of the penicillin concentration. However, when integrated density values were plotted vs. *c*_penicillin_, a higher sensitivity was found for ECL in comparison with fluorescence, arriving at quantitation ranges of 0.2–3.7 ppb and 6–119 ppb as well as LODs of 0.05±0.01 μg l^−1^ and 3.1±0.7 μg l^−1^ for ECL and fluorescence measurements, respectively. Moreover, ECL did not only outperform fluorescence again, but even more advantageously, the sensitivity on paper was improved with respect to that of the gAID system in suspension. A comparison of the images in Figure 5 a,b further reveals that the use of an electrode for excitation leads to a more homogeneous spot‐type signal.

Aiming to compare the signal amplification in suspension (see above, in conjunction with Figure 2) and on the strips, the ECL signal of **MMRAA@NPGF** at the two different penicillin concentrations used above, 1 and 7 ppb, was converted into a Ru(bpy)_3_^2+^ concentration by comparison with a calibration curve constructed from applying different known amounts of Ru(bpy)_3_^2+^ to **NPGF** and measuring ECL under identical conditions. The amplification factors found were 400 molecules of Ru(bpy)_3_^2+^ delivered on average per molecule of analyte for *c*_penicillin_=7 ppb and 1600 for 1 ppb of penicillin. The gAID system thus shows consistently a 4‐fold higher amplification on strip which is ascribed to a better concentration of the dye in the detection zone.

**Figure 6:**
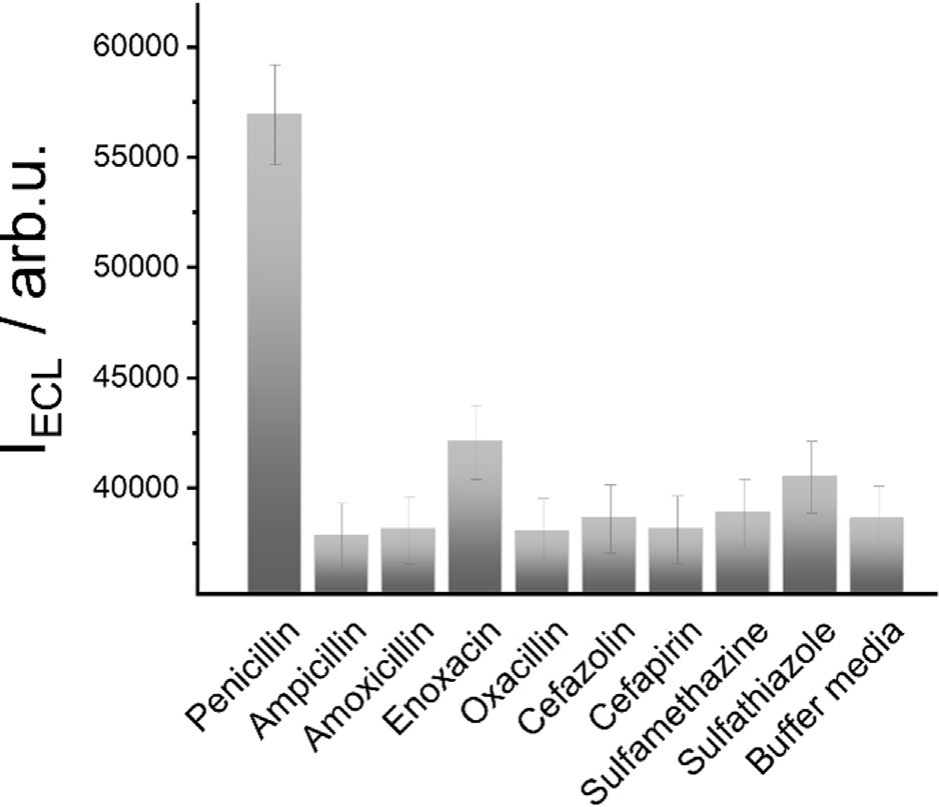
Integrated density of light emitted by Ru(bpy)_3_^2+^ released from **MMRAA** in presence of the antibiotics ampicillin, amoxicillin, enoxacin, oxacillin,cefazolin, cefapirin, sulfatiazole and sulfamethazine (250 ppb) in buffer (PB 10 mM; NBEA 25 mM; 75 %; pH 8) containing 25 % of milk on the strips after 2 min of reaction measured with the smartphone and ECL readout.

Finally, cross‐reactivities against other antibiotics were investigated by analysing several samples containing 250 ppb of ampicillin, amoxicillin, enoxacin, oxacillin, cefazolin, cefapirin, sulfamethazine and sulfathiazole with strips **MMRAA@NPGF** (for chemical structures, see Figure S19). Figure 6 reveals that only penicillin was able to significantly release the indicator from the pores with enoxacin and sulfathiazole showing a minor cross‐reactivity. The other antibiotics showed negligible dye release, similar to the blank release of the gAID material, in accordance with the reported selectivity of the aptamer.^[38a]^

For practical utility, the reproducibility of the materials production process and its long‐term storage are decisive. **MMRAA** showed unaltered performance in suspension when stored at 8 °C in a refrigerator under normal air atmosphere for 1 month, before blank release of dye became more pronounced. Concerning reproducibility, an error of 6–8 % found between replicates among a single batch of material increased to 7–15 % between assays using different batches of material, which is acceptable for such simple tests. When **MMRAA** was deposited on the strips, they showed unchanged performance over a storage period of ca. 3 months (same conditions as above), before the flow became slower, with errors of ca. 5 % between replicates and 6–12 % between strips with different batches. Upon storage for ≥11 months, the system still showed a very good performance with an only slightly reduced efficiency (LODs in lower ppb range), the aging of the coated fibres and/or wax barriers being most likely responsible for this. However, as the general performance remains unaltered, we tentatively assume that the stability of the material as well as the final test strip can be improved when packaged under CA (controlled atmosphere) conditions.

## Conclusion

In summary, the present work reported for the first time the favourable synergisms that can be obtained by combining electrogenerated chemiluminescence (ECL) detection on paper‐based test strips with gated indicator releasing materials. Toward a generic approach, covalently attached aptamers were used for the gating of the analyte‐induced release of an ECL reporter, endowing the system with a pronounced robustness that allowed for the direct determination of penicillin in a challenging matrix such as milk. The assay shows fast response times, good selectivity, and exceptional sensitivity, reaching an LOD of 50±9 ppt in an LFA with <5 min overall assay time. The recognition mechanism relies on the folding up of the aptamer upon penicillin binding, which leads to a disruption of the non‐covalent interactions that closed the pores and locked the ECL dye in them. Besides the intrinsic features of chemical signal amplification of gAID systems, optimization of ECL co‐reactant, SPE electrode and component concentration brought about a strong gain in sensitivity. For the strip assays, in particular the covalent modification of the paper fibres with hydrophilic yet uncharged PEG moieties and the additional immobilization of co‐reactant moieties allowed to outperform the solution assay. In comparison to other gAID systems for small‐molecule detection, the present approach performs significantly better than approaches that require a laboratory environment while reaching LODs such as 10.5 ppm for adenosine.^[44]^ Even the most sensitive laboratory‐based gAID assay for thrombin with an LOD of 0.13 ppb requires an analysis time of >120 min.^[45]^ Our system also outperforms other paper‐based ECL assays with readout via mobile communication and other handheld devices, being up to many orders of magnitude more sensitive.^[46]^ In addition, commercially available lateral flow tests for penicillin or other antibiotics relying on gold nanoparticle aggregation are also limited to ppb sensitivity.^[47]^ Keeping in mind that a large number of aptamers are reported in the literature for other types of analytes, this concept is easily generalizable, thus rendering it very attractive for point‐of‐care diagnostics, environmental or illicit drug analysis. Like demonstrated by us recently,^[29]^ the approach harbours a tremendous potential for low‐number multiplexing, which would require advancements in electrode and reporter design that are currently addressed in our laboratory. The successful detection of penicillin in milk suggests that such assays might indeed have a broad applicability especially for complex realistic matrices.

## Conflict of interest

The authors declare no conflict of interest.

## Supporting information

As a service to our authors and readers, this journal provides supporting information supplied by the authors. Such materials are peer reviewed and may be re‐organized for online delivery, but are not copy‐edited or typeset. Technical support issues arising from supporting information (other than missing files) should be addressed to the authors.

Supporting Information
