# Supplementary material for: Combining Electrochemiluminescence Detection with Aptamer‐Gated Indicator Releasing Mesoporous Nanoparticles Enables ppt Sensitivity for Strip‐Based Rapid Tests
Source: Angew Chem Int Ed Engl. 2021 Nov 9;60(50):26287–97. doi: 10.1002/anie.202110744 (PMC9298832; doi:10.1002/anie.202110744)
Supplement: Supplementary file 1 — Supporting Information [file ANIE-60-26287-s001.pdf]

Angewandte Chemie
Eine Zeitschrift der Gesellschaft Deutscher Chemiker

Supporting Information

Combining Electrochemiluminescence Detection with Aptamer-Gated Indicator Releasing Mesoporous Nanoparticles Enables ppt Sensitivity for Strip-Based Rapid Tests

Estela Climent and Knut Rurack**

anie_202110744_sm_miscellaneous_information.pdf

Author Contributions

E.C. and K.R. conceived the experiments. E.C. performed the experiments. E.C. and K.R. prepared the manuscript. Both authors discussed the results and commented on the manuscript.

Table of Contents

TABLE OF CONTENTS 2
GENERAL DESIGN AND PRINCIPLE OF OPERATION OF APTAMER-GATED INDICATOR DELIVERY SYSTEMS 3
1 ARCHITECTURE OF GATING BIOCHEMISTRY 3
EXPERIMENTAL PROCEDURES 5
2 GENERAL METHODS 5
3 SYNTHESIS AND CHARACTERIZATION OF MATERIALS 5
3.1 SYNTHESIS OF IRON OXIDE NANOPARTICLES (IO-NPS) 5
3.2 SYNTHESIS OF MAGNETIC MCM-41 MESOPOROUS SILICA NANOPARTICLES (MM) 6
3.3 SYNTHESIS OF DYE-LOADED (MMR) AND AMINATED MAGNETIC MESOPOROUS SILICA NANOPARTICLES (MMRA) 6
3.4 SYNTHESIS OF APTAMER-CAPPED, DYE-LOADED MAGNETIC MESOPOROUS SILICA NANOPARTICLES (MMRAA) 6
3.5 SYNTHESIS OF APTAMER- AND C-DNA CAPPED VARIANT MATERIAL MMRAAC 6
3.6 SYNTHESIS OF DYE-LOADED (MMR) AND CARBOXYLATED MAGNETIC MESOPOROUS SILICA NANOPARTICLES (MMRC) 6
3.7 SYNTHESIS OF APTAMER-CAPPED VARIANT MATERIAL (MMRCA) 6
4 CHARACTERIZATION OF MATERIALS 7
5 OPTIMIZATION OF ECL SYSTEM 11
5.1 CO-REACTANT SELECTION 11
5.2 ELECTRODE SELECTION 13
5.3 OPTIMIZATION OF CO-REACTANT CONCENTRATION 13
5.4 PH DEPENDENCE OF RU(BPY)$_3^{2+}$ ECL EMISSION 14
5.5 OPTIMISATION OF CO-REACTANT CONCENTRATION IN SUSPENSION ASSAY 15
6 SYNTHESIS AND CHARACTERIZATION OF PAPER STRIPS 17
6.1 SYNTHESIS OF IMMERSION-COATED MEMBRANES (NP)GF 17
6.2 SYNTHESIS OF SILANE-FUNCTIONALIZED MEMBRANES NPGF 17
6.3 INCORPORATION OF MATERIAL MMRAA INTO MEMBRANE NPGF (MMRAA@NPGF) 17
6.4 CHARACTERIZATION OF PAPERS 17
7 SMARTPHONE SETUP AS READOUT DEVICE FOR FLUORESCENCE DETECTION 19
8 OPTIMISATION OF STRIP-BASED ASSAYS 19
9 ADDITIONAL NOTES ON ANALYTICAL PERFORMANCE 21
9.1 LIMITS OF DETECTION 21
10 AUTHOR CONTRIBUTIONS 21
REFERENCES 22

General Design and Principle of Operation of Aptamer-Gated Indicator Delivery Systems

1 Architecture of gating biochemistry

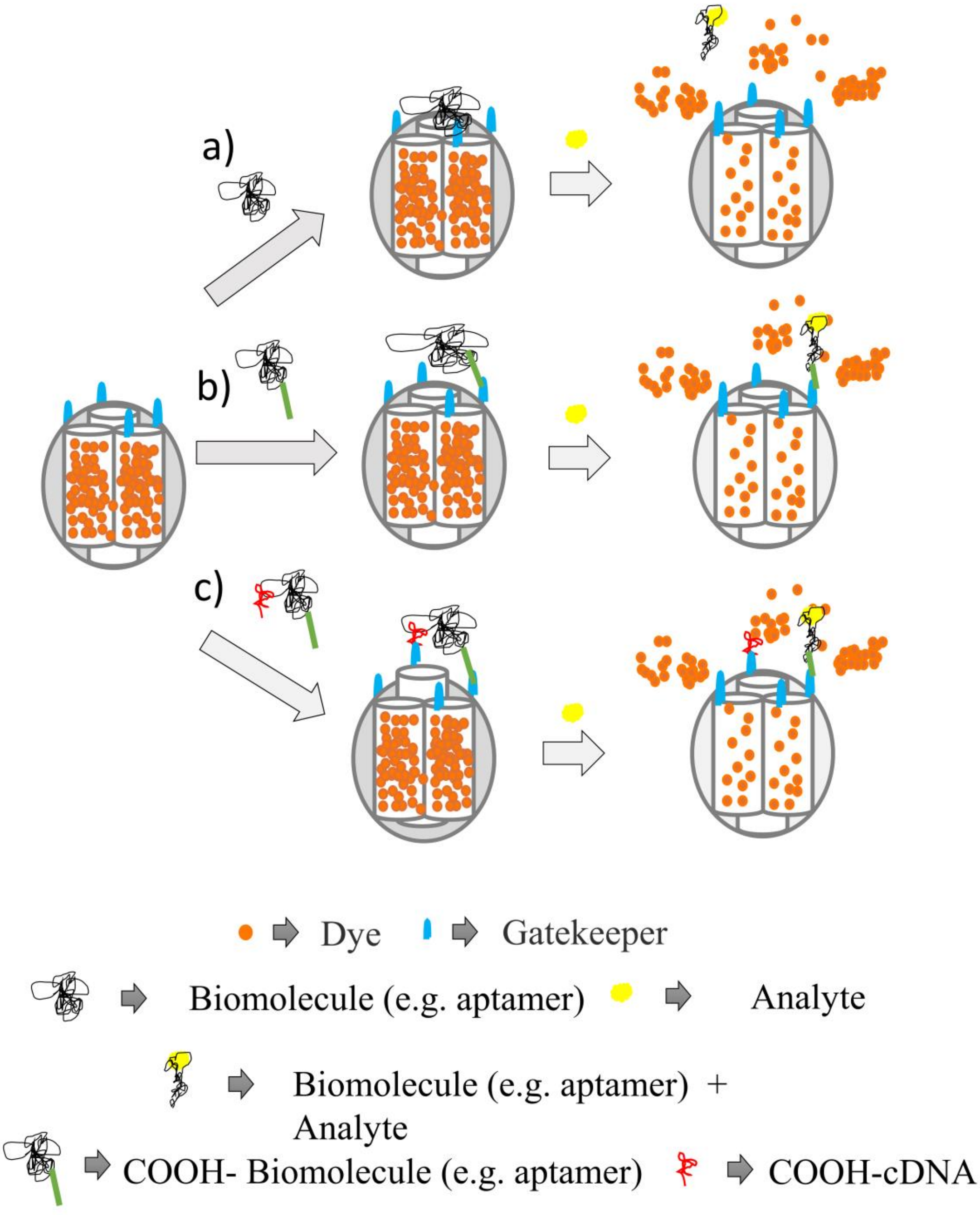

Scheme S1. Schematic representation of the preparation sequence and mode of operation of biomacromolecule-gated indicator delivery systems containing dye molecules as indicators in the pores, gatekeeper units covalently grafted to the surface of the mesoporous material and (a) biomolecules (e.g., aptamers) as caps that interact only non-covalently with the gatekeepers (e.g., amino groups), (b) biomolecules (e.g., aptamers) covalently grafted to the surface of the mesoporous material that cap the pore openings by non-covalent interaction with the gatekeepers (e.g., amino groups) and (c) a mixture of partially complementary biomolecular binders (e.g., aptamers and short c-DNA oligonucleotides), both covalently grafted to the surface of the mesoporous material and capping the pores by hybridization. In all the cases, the presence of the analyte produces a release of the dye due to complexation at the biomacromolecule's binding site, leading to refolding and desorption. Whereas in (a), the entire biomolecule is liberated and diffusing away, opening the pores, in (b) and (c) only the pore is opened.

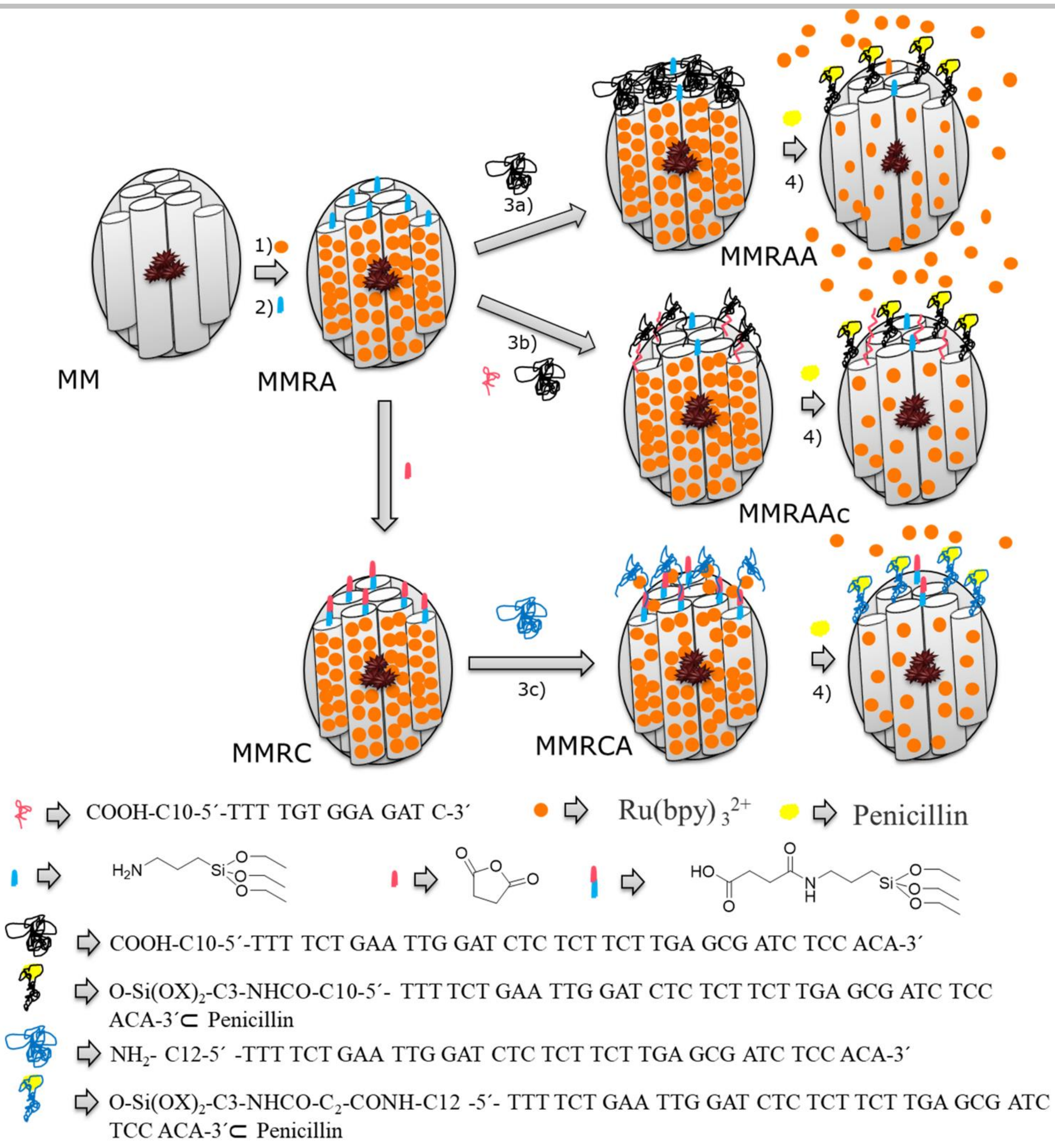

Scheme S2.- Schematic representation of the preparation sequence and mode of operation of the aptamer-gated indicator delivery (gAID) systems **MMRAAc** and **MMRCA** in comparison to **MMRAA**, based on 1) loading of Ru(bpy)$_3^{2+}$, 2) functionalisation of the outer surface with APTES moieties and derivatisation to carboxylic acid groups with succinic anhydride to obtain **MMRC**, 3a) grafting of the aptamer moieties to the surface via EDC/NHS coupling chemistry; 3b) grafting of the aptamer and c-DNA moieties to the surface via EDC/NHS chemistry; 3c) grafting of the aminated aptamer moieties to the surface via EDC/NHS chemistry and 4) release of the dye after binding of the corresponding analyte (penicillin).

Experimental Procedures

2 General methods

Chemicals and solvents were purchased from Sigma-Aldrich, ACBR, Merk and Metabion. Buffers were prepared with ultrapure reagent water, which was obtained by running demineralized water (by ion exchange) through a Milli-Q® ultrapure water purification system (Millipore Synthesis A10). Aptamers COOH-C10-5′-TTT TCT GAA TTG GAT CTC TCT TCT TGA GCG ATC TCC ACA-3′, NH2-C12-5′-TTT TCT GAA TTG GAT CTC TCT TCT TGA GCG ATC TCC ACA-3 and c-DNA oligonucleotide COOH-C10-5′-TTT TGT GGA GAT C-3′ were obtained from Metabion (HPLC purified and desalted). A standard solution of 100 µM in Mili-Q water was prepared and stored as well as aliquoted and stored at 8 °C for short periods (1 month) and at –28°C for long-term periods.

Absorption, fluorescence and electrochemiluminiscence spectroscopy, elemental analysis, thermogravimetric analysis (TGA), transmission electron microscopy (TEM), high-angle annular dark-field scanning transmission electron microscopy with energy dispersive X-ray microanalysis (HAADF-STEM-EDX), scanning electron microscopy with energy dispersive X-ray microanalysis (SEM-EDX) and N_2 adsorption-desorption were employed to characterize the synthesized materials and test their behaviour towards the corresponding analytes. UV-vis spectra were measured with a Specord 210plus from Analytik Jena. Cyclic voltammetry, linear sweep voltammetry and amperometry measurements, in solution and on glass fibre membranes, were performed with a spectroelectrochemiluminescence instrument for use with screen-printed electrodes (SpectroECL) from Metrohm-Dropsens. A portable potentiostat (Sensismart; Palmsens) able to be connected to a smartphone was also employed for measurements with a portable device. Fluorescence measurements were carried out on a Fluoromax4 from HORIBA Scientific. Thermogravimetric analyses were carried out on a STA7200 thermobalance (Hitachi High-Tech Analytical Science), using in a first step a nitrogen atmosphere (80 mL min^{-1}) with a heating program consisting of a ramp of 10 °C min^{-1} from 25 °C to 600 °C and in a second step an oxidizing atmosphere (air, 80 mL min^{-1}) from 600 °C until 1000 °C with a heating program consisting of a ramp of 10 °C min^{-1}. Elemental analyses were carried out with a Euro EA-Elemental Analyser from HEKAtech. TEM and STEM images as well as energy-dispersive X-ray spectroscopy (EDX) analyses on the particles were obtained with a Talos F200S scanning/transmission electron microscope from FEI. SEM images and energy-dispersive X-ray spectroscopy (EDX) analyses on the sensing membranes were performed on a Zeiss Supra 40 single-unit transmission set-up equipped with a high-resolution cathode (Schottky field emitter) and an In-Lens SE secondary electron detector used in the high-resolution mode, whereas EDX spectra were measured with a Si(Li) EDS detector (Thermo Scientific) or an XFlash® SDD detector (Bruker), both having an active area of 10 mm^2. N_2 adsorption-desorption isotherms were recorded with a Micromeritics ASAP2010 automated sorption analyzer. The samples were degassed for 3 h at 200 °C in a vacuum. The specific surface areas were calculated from the adsorption data in the low-pressure range using the Brunauer-Emmett-Teller (BET) model. Pore sizes were determined following the Barrett-Joyner-Halenda (BJH) method. Small- and wide-angle X-ray scattering (SAXS/WAXS) measurements were performed on a customized Xeuss 2.0 (Xenocs). X-rays were generated from a microfocus X-ray tube with a copper target, and a multilayer optic was employed to parallelize and monochromatize the beam to the Cu-Kα wavelength of 0.1542 nm. Data collection was performed using an in-vacuum Eiger 1M detector (Dectris, Switzerland) which was placed at multiple distances between 51–2507 mm from the sample. The resulting data has been processed and scaled to absolute units using the DAWN software package according to standardized procedures.[1] For the smartphone-based approach, a 3D-box was printed with black PLA using an Ultimaker 3 printer. LEDs and optical filters were purchased from Thorlabs. Photographs were taken with a Samsung Galaxy S7 and values retrieved from images via the integrated density with the software ImageJ, i.e., the product of mean grey value of the red channel, and the selected area *a* (in square pixels).

3 Synthesis and characterization of materials

*3.1 Synthesis of iron oxide nanoparticles (**IO-NPs**)*

Iron oxide nanoparticles were synthesized according to a modified procedure reported previously[2] by the reverse co-precipitation method, employing iron(II) chloride and iron(III) chloride as the iron precursors and sodium hydroxide (NaOH) as the base. For that purpose, 3.75 ml of NaOH 10 M and 45 ml of water were in a first instance de-aerated with Argon during 30 min at room temperature. Then, a solution of 5 ml of deionized water containing 2.92 g $FeCl_3 \cdot 6H_2O$ and 1.075 g $FeCl_2 \cdot 4H_2O$ was added to an offset funnel before dropwise addition during 10-15 min and leaving it for 1 h at room temperature (rt) under argon atmosphere, yielding the iron oxide nanoparticles. Finally, 50 ml of trisodium citrate solution (0.3 M) was added and then stirred for 30 min at rt, obtaining as a result a stable, citrate-coated iron oxide nanoparticle dispersion. The ultrafine magnetic particles were precipitated with acetone and the supernatant was decanted with the help of a magnet. Particles were resuspended with 100 ml of water to a final concentration of 2.6% (w/v %) of particles.

*3.2 Synthesis of magnetic MCM-41 mesoporous silica nanoparticles (**MM**)*

Magnetic mesoporous MCM-41 nanoparticles (MMSN) were synthesized as reported previously.[3] A solution of 11.5 ml of the iron oxide nanoparticles prepared before (**IO-NPs**; 2.6%, 300 mg) were mixed with 480 ml of deionized water and the suspension was sonicated in an ultrasonic bath for 15 min. Then, *n*-cetyltrimethylammonium bromide (CTABr, 1.00 g, 2.74 mmol) and 3.5 ml of 2 M NaOH in deionized water were added to the suspension, and the suspension was sonicated for another 15 min. Afterwards, the suspension was heated to 80 ºC, and tetraethylorthosilicate (TEOS; 5.00 ml, 2.57×10^{-2} mol) was added dropwise to the suspension. The mixture was stirred for 2 h at 80°C, yielding a brown precipitate. The solid product was centrifuged, washed with deionized water and ethanol (2:1) and dried at 60 ºC (MMSN as-synthesized). Removal of the template was achieved by calcination with a heating program consisting of a ramp of 2 °C min^{-1} from 25°C to 550°C and followed by a constant heating at 550 °C for 5 h, yielding the calcinated porous material **MM** (MMSN calcinated).

*3.3 Synthesis of dye-loaded (**MMR**) and aminated magnetic mesoporous silica nanoparticles (**MMRA**)*

With the aim of loading the maximum amount of dye into the calcinated MMSN scaffold, 10 ml of a solution of tris(bipyridine)ruthenium(II) chloride ($Ru(bpy)_3^{2+}$; 6.3 mmol L^{-1}) prepared in acetonitrile (MeCN) was added to 100 mg of **MM**. The suspension was stirred for 24 h at rt, yielding **MMR**. Subsequently, 3-aminopropyltriethoxysilane (APTES; 225 µl; 10 mmol g^{-1} solid) was added to the suspension, and the resulting mixture was stirred for 5.5 h at rt. The solid **MMRA** (containing $Ru(bpy)_3^{2+}$ in the inner pore voids and amino groups preferentially located on the outer particle surface) was obtained after centrifugation (5 min at 6000 rpm), washing with acetonitrile (5 ml), further centrifugation, and drying at 30 °C in a vacuum for 2 h.

*3.4 Synthesis of aptamer-capped, dye-loaded magnetic mesoporous silica nanoparticles (**MMRAA**)*

The aptamer for the detection of penicillin, COOH-C10-5′-TTT TCT GAA TTG GAT CTC TCT TCT TGA GCG ATC TCC ACA-3′,[4] was grafted covalently through its residual carboxylic acid group to particles **MMRA** containing amino groups on the surface via an active-ester method; following a modified procedure reported by us previously.[5] For that purpose, 100 µl of a freshly prepared 1 % (1-ethyl-3-(3-dimethylaminopropyl)carbodiimide hydrochloride) (EDC) solution in 2-(*N*-morpholino)ethanesulfonic acid (MES) buffer (pH 5, 0.1 M), containing $Ru(bpy)_3^{2+}$ (10 mg ml^{-1}) and 100 µl of a 1 % *N*-hydroxysulfosuccinimide (Sulfo-NHS) solution in MES buffer, were added to 20 µl of a 100 µM standard solution of the penicillin aptamer. After stirring for 15 min at rt, the solution was added to a suspension of 2 mg of **MMRA** in 100 µl of MES buffer (pH 5, 0.1 M), again containing $Ru(bpy)_3^{2+}$ (10 mg ml^{-1}). The mixtures were left to react in a thermomixer (1000 rpm) at 45 °C overnight. Particles **MMRAA** were isolated from the supernatant with the help of a magnet, washed once with 500 µl of phosphate buffer (PB, 10 mM, pH 8) and resuspended in 1 ml of PB, splitting the suspension in fractions of 100 µl and storing them in the refrigerator at 8 °C. These suspensions were stable for a period of one month (for more details on stability issues, see Section 9).

*3.5 Synthesis of aptamer- and c-DNA capped variant material **MMRAAc***

MMRAc was prepared following a similar procedure as for **MMRAA**, only that a mixture consisting of 20 µl of 100 µM standard solution of the penicillin aptamer and 20 µl of 100 µM standard solution of c-DNA oligonucleotide COOH-C10-5′-TTT TGT GGA GAT C-3′ were employed simultaneously during the preparation of the active ester.

*3.6 Synthesis of dye-loaded (**MMR**) and carboxylated magnetic mesoporous silica nanoparticles (**MMRC**)*

With the aim to functionalize the external surface of **MMRA** with carboxylic acid moieties, 25 mg **MMRA** were suspended in1.5 mL of Ethanol. Subsequently, 100 µl of a solution of succinic anhydride (1% w/v) were added to the suspension, leaving the suspension stirring for 20 h at 45 °C. The solid **MMRC** (containing $Ru(bpy)_3^{2+}$ in the inner of the pore voids and carboxylic acid groups preferentially located on the outer particle surface) was obtained after centrifugation (5 min at 6000 rpm), washing 3 times with 1.5 mL with deionized water and ethanol (1:1), further centrifugation, and drying at 30 °C in a vacuum for 2 h.

*3.7 Synthesis of aptamer-capped variant material (**MMRCA**)*

The aminated aptamer for the detection of penicillin, NH_2-C12-5′-TTT TCT GAA TTG GAT CTC TCT TCT TGA GCG ATC TCC ACA-3′,[4] was grafted covalently through its residual amino group to particles **MMRC** containing carboxy groups on the surface via an active-ester method; following a modified procedure reported above. For that purpose, 100 µl of a freshly prepared 1 % (1-ethyl-3-(3-dimethylaminopropyl)carbodiimide hydrochloride) (EDC) solution in 2-(*N*-morpholino)ethanesulfonic acid (MES) buffer (pH 5, 0.1 M), containing $Ru(bpy)_3^{2+}$ (10 mg ml^{-1}) and 100 µl of a 1 % *N*-hydroxysulfosuccinimide (Sulfo-NHS) solution in MES buffer, were added to

a suspension of of 2 mg of **MMRC** in 100 µl of MES buffer (pH 5, 0.1 M), again containing $Ru(bpy)_3^{2+}$ (10 mg ml^{-1}). After stirring for 15 min at rt, 20µl of penicillin aptamer was added to a suspension. Mixtures were left to react in a thermomixer (1000 rpm) at 45 °C overnight. Particles **MMRCA** were isolated from the supernatant with the help of a magnet, washed once with 500 µl of phosphate buffer (PB, 10 mM, pH 8) and resuspended in 1 ml of PB, splitting the suspension in fractions of 100 µl and storing them in the refrigerator at 8 °C in a similar way to the one reported above.

4 Characterization of materials

All solids were characterized by standard procedures. The presence of the mesoporous structure was confirmed with nitrogen adsorption-desorption isotherms, scanning electron microscopy (SEM) and transmission electron microscopy (TEM) analysis, whereas the respective contents of aptamer, APTES and $Ru(bpy)_3^{2+}$ in and on the materials were estimated from elemental analysis, thermogravimetry and absorbance measurements.

First, small and wide-angle X-Ray scattering (SAXS/WAXS) measurements were carried out with **IO-NPs** and **MM**. WAXS patterns of the **IO-NPs** showed diffraction peaks at higher scattering *q* vector values that can be indexed to the cubic array of a mixture of magnetite (Fe_3O_4) and maghemite (γ-Fe_2O_3 mixtures),[6] showing the diffraction peaks corresponding to the (220), (311), (400), (422), (511), (440), (620) and (533) crystallographic planes (Figure S1a). SAXS and WAXS patterns of the magnetic mesoporous nanoparticles **MM** showed the typical diffraction peaks at lower *q*-values, distinctive of a mesoporous MCM-41-type material corresponding to (100) and (110), a broad band between $10 < q < 20$ attributed to the presence of tiny amounts of amorphous silica and also the characteristic reflections of the **IO-NPs** at higher *q* values (Figure S1b). Taking a look to the diffraction peaks in more detail, several diffraction peaks indexed to hematite (α-Fe_2O_3) were observed in the WAXS pattern, which suggests that some magnetite or maghemite in **IO-NPs** have converted to hematite in **MM** nanoparticles due to the calcination treatment for the removal of the surfactant.[7] Furthermore, comparing the diffraction peaks of **IO-NPs** and **MM** we observed that the width of the diffraction peaks became much narrower, which suggested that the calcination treatment of **MM** nanoparticles induced the formation of larger crystals of magnetite or maghemite. In both cases, lattice cell parameters of a = 0.844 ± 0.006 nm and 4.59 ± 0.06 nm for **IO-NPs** and **MM** nanoparticles were determined, respectively.

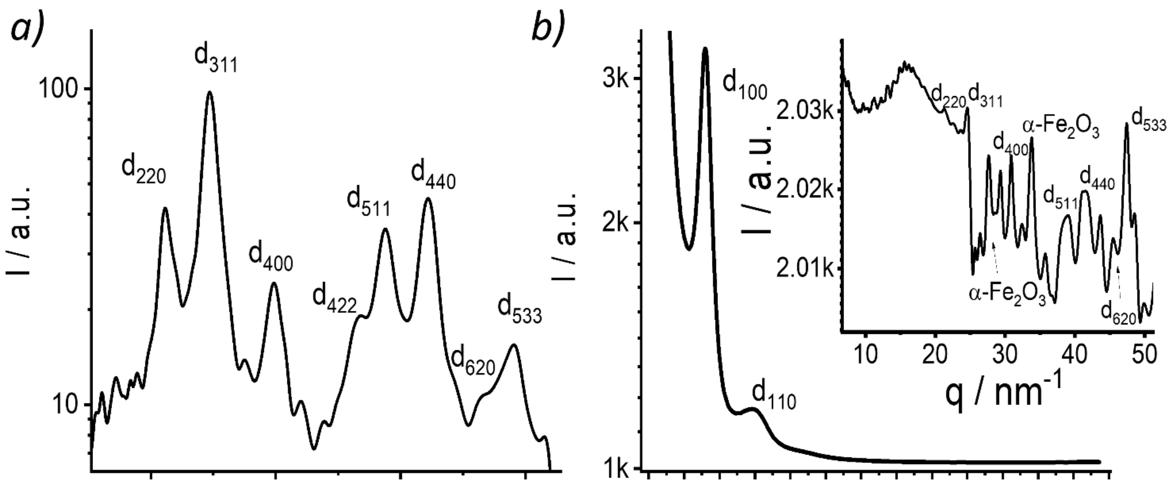

Figure S1. a) Small- and wide-angle X-ray scattering patterns of a) **IO-NPs** and b) **MM** nanoparticles. Inset in b) corresponds to the WAXS patterns of **MM** in the regions between $10 < q < 50$ nm^{-1}.

SEM and TEM images revealed that the calcinated silica mesoporous nanoparticles **MM** were obtained as spherical particles with radii ranging between 70–110 nm, with an average size of 205 ± 34 nm, having encapsulated a certain number of magnetic nanoparticles with diameters of 6.5 ± 1.1 nm mostly in the centre of the particles (Figure S2). The TEM images in Figure S2c also show the mesoporosity of this type of particles, which was slightly more disordered in comparison to conventional MCM-41, tentatively ascribed to the presence of magnetic iron oxide particles. Figure S2c also shows that the porous structure remains in capped material **MMRAA** remained intact after the loading of the dye and the functionalisation of the material.

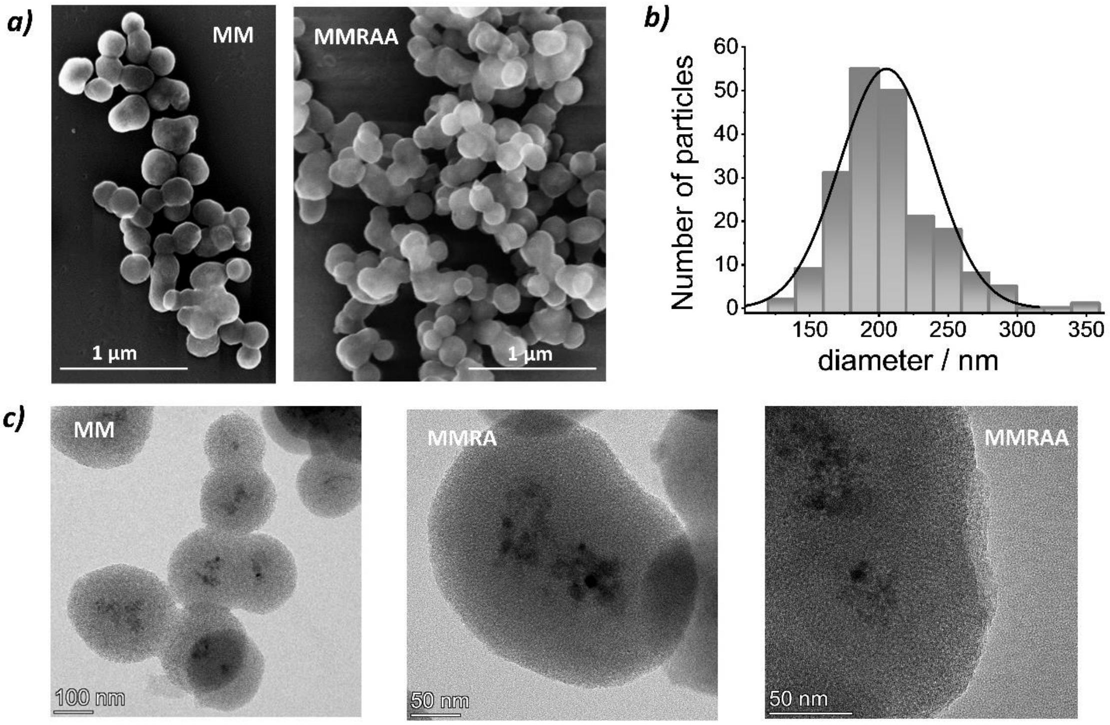

Figure S2. a) SEM images of nanoparticles **MM** and **MMRAA**: b) Corresponding size distribution of **MM** (200 particles analysed). C) TEM images of **MM**, **MMRA** and **MMRAA** nanoparticles, showing the typical porosity of the MCM-41 mesoporous matrix (black and white dots) and the magnetic iron oxide nanoparticles located in the centre of the particles.

Nitrogen adsorption-desorption isotherms of the calcinated **MM** nanoparticles are shown in Figure S3. The presence of an adsorption step at intermediate P/P_0 values (0.25–0.35) related to the nitrogen condensation inside the mesopores showed a typical type-IV isotherm. The absence of a hysteresis loop, in combination with the narrow BJH pore distribution, indicated the presence of uniform cylindrical mesopores. A pore diameter of 2.4 nm and a pore volume to 0.67 $cm^3\ g^{-1}$ were estimated by applying the BJH model on the adsorption branch of the isotherm. Furthermore, the application of the BET model resulted in a value of 1009 $m^2\ g^{-1}$ for the total specific surface area. From the SAXS, porosimetry and TEM studies, and having in mind the lattice cell parameter (4.59 ± 0.06 nm) and the pore diameter (2.4 nm), a value for the wall thickness of 2.18 nm was derived. A second adsorption step at a higher relative pressure (P/P_0>0.9) was also observed, which corresponds to the filling of the large voids between the particles and can be ascribed to textural porosity. N_2 adsorption-desorption isotherms of **MMRAA** could not be obtained because of the large amount of material needed, but a decrease of the pore volume and also of the specific surface area due to filling of the pores with the dye and functionalisation of the surface with aptamer and amino moieties is expected.

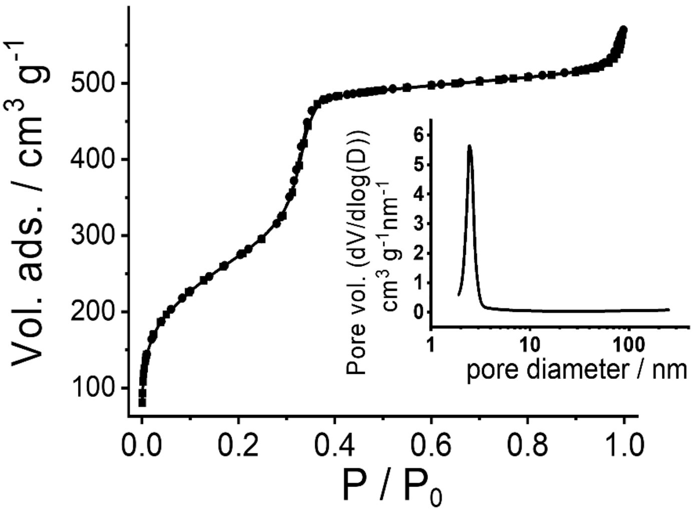

Figure S3. a) N_2 adsorption-desorption isotherm for calcined silica mesoporous nanoparticles **MM**. Inset: Corresponding pore size distribution

The contents of APTES, carboxylic acid groups and $Ru(bpy)_3^{2+}$ dye in **MMRA** and **MMRC** were determined by elemental analysis and thermogravimetric measurements. Furthermore, the amount of $Ru(bpy)_3^{2+}$ after the aptamer capping in **MMRAA**, **MMRAAc** and **MMRCA** was determined indirectly from the difference between the amount measured in **MMRA** and the amount washed out into the aqueous washing fractions after the capping process. Finally, the amount of aptamer on the materials was estimated from spectrophotometric measurements using a standard addition method and measuring the increase in absorbance at 260 nm. All data are collected in Table S1.

Table S1. Contents of APTES, carboxylic acid groups (COOH), $Ru(bpy)_3^{2+}$ and aptamer groups in **MMRA**; **MMRC, MMRAA, MMRAAc** and **MMRCA**.

Solid	APTES	COOH	$Ru(bpy)_3^{2+}$	Aptamer
	mmol g^{-1} solid	mmol g^{-1} solid	mmol g^{-1} solid	µmol g^{-1} solid
**MMRA**	1.03 ± 0.05	-	0.26 ± 0.03	-
**MMRAA**	1.03 ± 0.05	-	0.21 ± 0.02	0.7 ± 0.3
**MMRAAc**	1.03 ± 0.05	-	0.18 ± 0.02	0.6 ± 0.3
**MMRC**	-	0.95 ± 0.03	0.21 ± 0.01	
**MMRCA**	-	0.95 ± 0.03	0.16 ± 0.02	0.4 ± 0.2

Zeta potential measurements and EDX analysis from TEM and STEM were used to determine the presence of the aptamer and qualitatively estimate the average amount of Fe, C, N, P, Ru and Si for the materials **MM**, **MMRA** and **MMRAA**. Zeta potential measurements were performed in water buffered at different pH (MES 100 mM pH 5, PB 10 mM pH 8) and in neat water at pH 7, Figure S4. In all cases, **MM** presented a negatively charged surface due to the presence of silanol groups. The relative negative charge was dependent on pH, becoming less negative with more acidic pH. Addition of amino groups to the surface of **MMRA** produced a positively charged surface, showing a corresponding pH dependence with a reduction in positive charge as the pH increases, due to the partial conversion of ammonium into amino groups when pH increases. Covalent grafting of the aptamer moieties on the material produced again a negatively charged particle at basic pH, which became less negative as the pH was reduced and eventually even slightly positive at pH 5. The reduction of positive charge of the aminated particles can be explained by partial conversion of amino groups into amido groups upon aptamer attachment. However, given the size of the aptamers, most amino groups will remain available for protonation as surface coverage of micro- and nanoparticles with DNA is much less dense than with amino groups (e.g., a factor of >600 in functionalisation density has recently been found for 18mer DNA vs. APTES-originating amino groups, ref. [8]). This slight reduction in protonatable amino groups can thus most likely not only be the cause for the observed zeta potential changes. Instead, we tentatively ascribe a larger effect stemming from electrostatic interaction of negatively charged phosphate groups on the aptamer's backbone with the surface amino groups, reducing their influence more pronouncedly. After addition of 2 ppm of penicillin, a small displacement to more positive zeta potential values were again observed especially at neutral pH at which binding of penicillin by the aptamer is most efficient. Interaction of penicillin with the aptamer leads to a conformational rearrangement, the aptamer folds up and the shielding of the surface amino groups through non-covalent electrostatic interactions is reduced, entailing an opening of the pores.

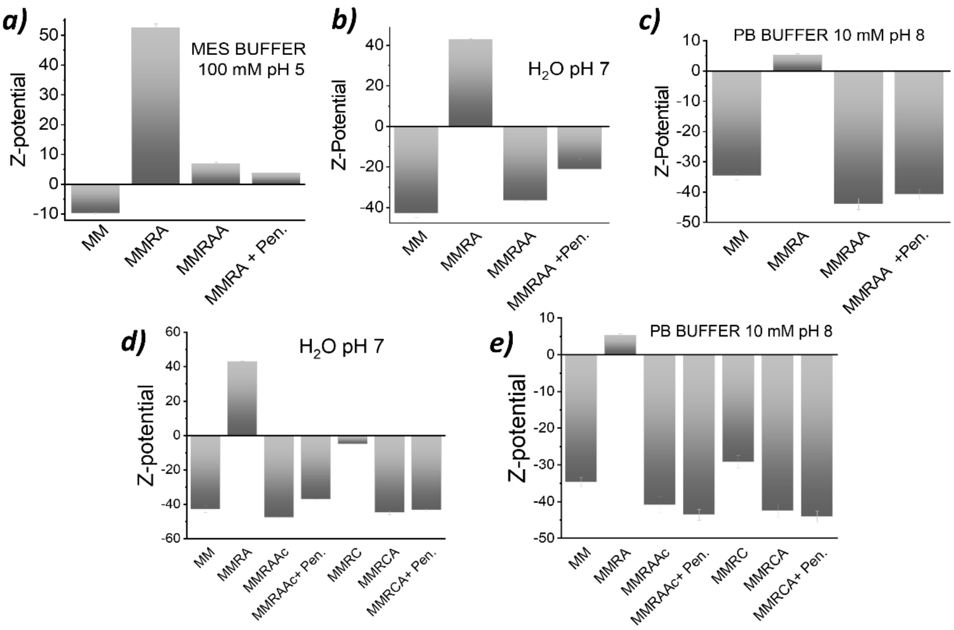

Figure S4. a)-c) Zeta potential values of materials **MM**, **MMRA**, **MMRAA** and **MMRAA** in the presence of 2 ppm of penicillin in a) MES buffer (100 mM) at pH 5, b) H_2O at pH 7 and c) PB buffer (10 mM) at pH 8. d)-e) Zeta potential values of materials **MM**, **MMRA**, **MMRC**, **MMRAAc** and **MMRCA** in the presence of 2 ppm of penicillin in d) H_2O at pH 7 and e) PB buffer (10 mM) at pH 8.

The EDX analyses from TEM and STEM are shown in Figure S5. The loading of **MMRA** with the dye and the functionalisation with APTES moieties leads to an increase in N and C, whereas after the incorporation of the aptamer, a significant increase of the phosphorous content (due to the chemical composition of nucleotides) was observed. In addition, a small decrease in the content of Ru in **MMRAA** in comparison with **MMRA** was found, which can be related to a loss of dye during the washing steps after the aptamer coupling. Despite the trends observed, it is important to note that the values in Table S2 are more qualitative than quantitative, considering that the uncertainty of measurements is considerably high (in some cases more than 25%) due to the light elements analysed and the low amounts present, and also because of partial damage of the materials during scanning with a high-energy beam.

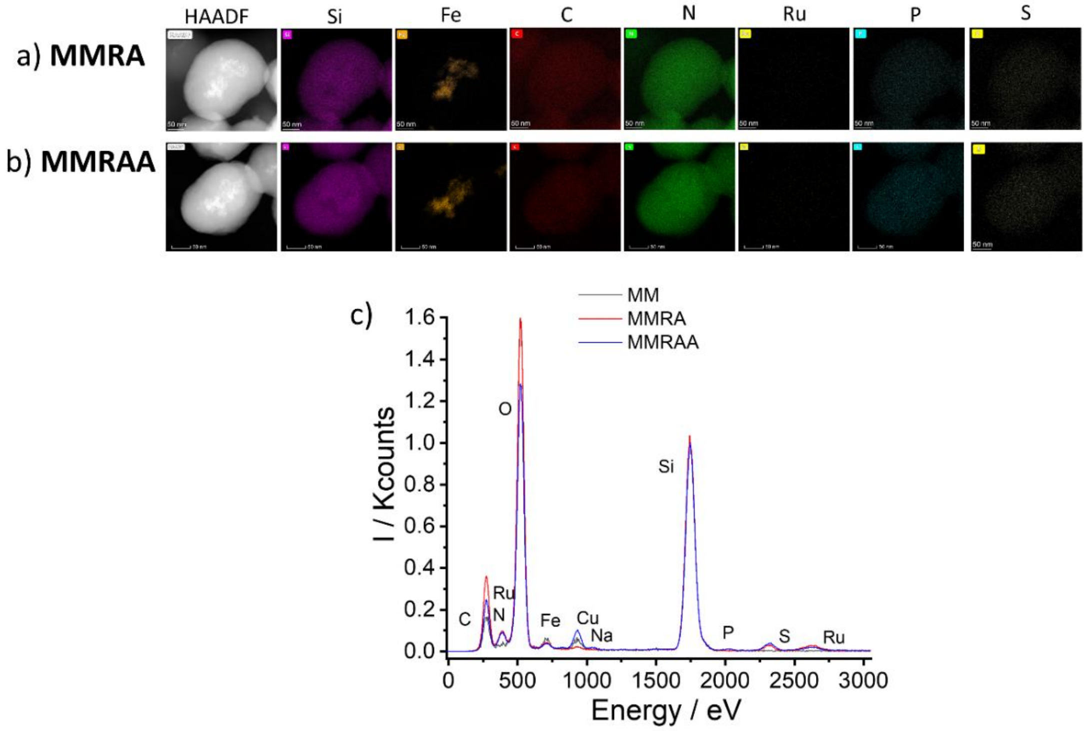

Figure S5. High-angle annular dark-field STEM images and corresponding EDX element maps of Si, Fe, C, N, Ru P and S for a) **MMRA** and b) **MMRAA** materials. c) Corresponding EDX intensities (normalised to Si) of the different materials **MM**, **MMRA** and **MMRAA**

Table S2. Amounts of Fe, C, N, P and Ru (in mmol g SiO_2^{-1}) estimated through EDX measurements.

	**mmol Fe g^{-1} SiO$_2$^{a}**	**mmol C g^{-1} SiO$_2$^{a}**	**mmol N g^{-1} SiO$_2$^{a}**	**mmol P g^{-1} SiO$_2$^{a}**	**mmol Ru g^{-1} SiO$_2$^{a}**
**MM**	0.90 ± 0.20	6.36 ± 3.60	-	-	-
**MMRA**	0.89 ± 0.10	13.27 ± 3.57	1.34 ± 0.27	0.03 ± 0.01	0.14 ± 0.02
**MMRAA**	0.84 ± 0.10	27.70 ± 6.10	2.25 ± 1.30	0.13 ± 0.02	0.10 ± 0.02
**MMRAAc**	0.87 ± 0.52	30.78 ± 4.78	2.73 ± 0.25	0.08 ± 0.02	0.10 ± 0.03
**MMRC**	0.75 ± 0.30	23.87 ± 4.67	2.20 ± 1.10	-	0.09 ± 0.03
**MMRCA**	0.73 ± 0.10	23.10 ± 2.78	2.60 ± 2.84	0.05 ± 0.02	0.06 ± 0.02

[a] The quantification obtained through SEM-EDX of the elemental composition is a very rough estimation due to the light elements C, N, P and Si analyzed and also due to the low amounts present on the materials. Due to this, data obtained can only be considered to compare the results obtained between the different materials analyzed.

5 Optimization of ECL system

ECL was generated by linear sweep voltammograms (LSV) scanning from 0.1 to 1.3 or 1.6V V with scan rates of 20 mV s^{-1}, whereas ECL spectra were recorded with an integration time of 5 s. For the detection of the ECL generated by the smartphone setup, amperometry measurements were performed to collect the maximum ECL light as a function of the time, because it allowed the greatest control over the timing and duration of the reaction. For that purpose, after introduction of the sample solution or the sensing strip, the potential was increased stepwise from 0 to 1.1 V in 0.5 s and maintained at 1.1 V for 10 s. On application of the potential, an image of the ECL emission in the paper was immediately acquired using a Samsung Galaxy S7 with an ISO of 800 and a time of exposition of 10 s, the mobile phone acting as the photodetector. The images were analyzed using the software ImageJ, which allowed to separate the pixels into red, green, and blue and generate the total values for each of the intensities. The integrated density of the red pixels was evaluated to control the response of the sensing material as a function of the concentration. ECL enhancements as a function of the concentration were determined by the difference of the ECL intensity at a given concentration of $Ru(bpy)_3^{2+}$ and the ECL intensity in absence of the analyte divided by the ECL intensity in absence of the analyte. Before each ECL determination, the working electrode was thoroughly rinsed with water.
With respect to the reusability of electrodes, especially when targeting matrices like milk, potential SPE performance losses during successive measurements was also evaluated. For that purpose, the ECL of solutions containing 15 µM of $Ru(bpy)_3^{2+}$ in milk (25%) diluted with buffer (PB 10 mM; NBEA 5 mM; 75%; pH 8) was measured repetitively (12 times) by amperometry using a single SPE electrode and washing it in between only with water as described before. A high concentration of ECL dye was chosen, to evaluate also small efficiency losses. Whereas the intensity of the signal was constant during the first 5–7 measurements, the ECL intensity dropped somewhat by ca. 13% after 10–12 measurements, which is acceptable for point-of-need use outside of a laboratory.

5.1 Co-reactant selection

It is known that the ECL signal from $Ru(bpy)_3^{2+}$ is relatively low in aqueous environments but can be enhanced by the use of co-reactants.[9] For this purpose, 12.5 mM solutions of the co-reactants 2-(dibutylamino)ethanol (DBAE), *N*-butyldiethanolamine (NBEA) and tri-n-propylamine (TPA) in phosphate buffer (PB; 10 mM, pH 8) were spiked with $Ru(bpy)_3^{2+}$ to a final concentration $c_{Ru(bpy)3}$ = 1 mM in all cases. A solution containing only PB was also spiked with $Ru(bpy)_3^{2+}$ as a reference. 50 µl of the solutions were deposited in the ECL cell of the spectraECL device used for measurements, and a linear sweep voltammetry (LSV) scan was registered, employing a screen-printed electrode (SPE) with carbon as the working and the counter electrode and silver as the reference electrode (C110; see Table S3). Figure S6 reveals that the best ECL signal enhancement was found for NBEA as the co-reactant. DBAE and NBEA showed stronger ECL signal intensity in comparison with TPA, despite the fact that higher applied potentials were needed to produce the corresponding ECL signal (1.3 V for NBEA, 1.2V for DBAE and 1.1 V for TPA, respectively, see Figure S6a). ECL of $Ru(bpy)_3^{2+}$ solution containing no co-reactant was only able to produce a slight ECL enhancement, reinforcing the importance of the employment of a co-reactant in the assays (inset Figure S6b). The same experiment was repeated with $Ru(bpy)_3^{2+}$ solutions of 200 µM, and in that case, NBEA showed stronger ECL in comparison to DBAE and TPA. Due to this, we choose NBEA as co-reactant for further experiments.

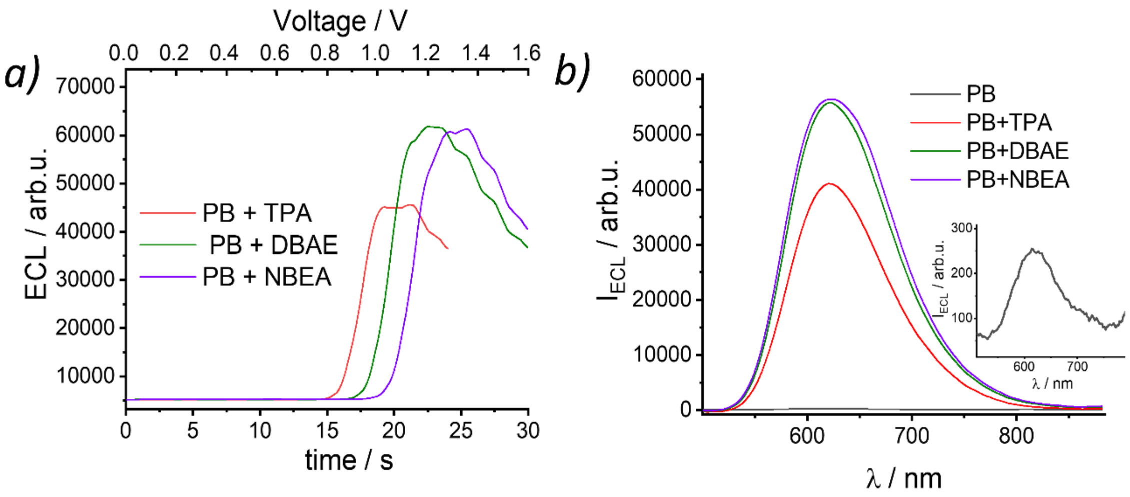

Figure S6. a) ECL observed as a function of the voltage and the time under linear sweep voltammetry (LSV) assay conditions. b) Corresponding ECL spectra obtained at 1.3V.

Table S3. Type of Working electrode (WE), counter electrode (CE) and reference electrode (RE) of the screen-printed electrodes (SPE) tested.

SPE Electrode	Working Electrode	Counter Electrode	Reference Electrode	Voltage applied for ECL max. / V
**220BT**[a]	Gold	Gold	Silver	0.85
**220AT**[a]	Gold	Gold	Silver	1.30
**250AT**[a]	Gold	Platinum	Silver	1.10
**AUTR10**	Gold	Carbon	Silver	0.85
**C110**	Carbon	Carbon	Silver	1.30
**DS850**	Carbon	Platinum	Silver	1.40
**COTE10**	CWCNT	Carbon	Silver	0.85
**P10**	PEDOT	Carbon	Silver	-

[a] Electrodes screen-printed with high (AT) and low (BT) temperature curing inks

The mechanism for the production of ECL using $Ru(bpy)_3^{2+}$ and NBEA is similar to the one observed for TPA and also for DBAE, and is proposed to be as follows:[10]

$$Ru(bipy)_3^{2+} - e^- \longrightarrow Ru(bipy)_3^{3+} \quad (1)$$

$$NBEA - e^- \longrightarrow NBEA^{\bullet +} \quad (2)$$

$$NBEA^{\bullet +} \longrightarrow NBEA^{\bullet} + H^+ \quad (3)$$

$$Ru(bipy)_3^{3+} + NBEA^{\bullet} \longrightarrow Ru(bipy)_3^{2+*} \quad (4)$$

$$Ru(bipy)_3^{2+*} \longrightarrow Ru(bipy)_3^{2+} + h\nu \quad (5)$$

$Ru(bipy)_3^{2+}$ and the amino group of NBEA are oxidized at the surface of the electrode to form $Ru(bipy)_3^{3+}$ (1) and the radical cation $NBEA^{\bullet+}$ (2). Subsequently the deprotonation of the radical $NBEA^{\bullet+}$ produces the reducing $NBEA^{\bullet}$ radical (3), which reduces the $Ru(bpy)_3^{3+}$ to the excited state, $Ru(bpy)_3^{2+*}$ (4), allowing the light emission with a band centered at 620 nm through its relaxation to the ground state (5).

5.2 Electrode selection

Different electrode materials were screened for optimum ECL yield, i.e., SPEs with the working electrodes fabricated from gold, carbon and poly(3,4-ethylenedioxythiophene) polymer (PEDOT) were investigated. The choice of the right electrode material is crucial to achieve best electron transfer for the ECL reaction, since the direct oxidation of the co-reactant at the surface of the electrode influences the ECL efficiency significantly.[11] SPEs are very convenient as they can dispense with the need to clean the electrode surface after each use by polishing, yet the quality of commercially available SPEs is related to the screen-printing inks and polymeric binders employed.[12] LSV scans of a solution of 10 µM of $Ru(bpy)_3^{2+}$ and 25 mM of co-reactant NBEA in PB (10 mM) were registered with the electrodes containing a ceramic (220AT, 220BT, 250AT, C110, DS850; see Table S3) or a thin plastic transparent substrate (AUTR10, COTE10 and P10; see Table S3). As can be seen in Figure S7, a strong reddish-orange ECL signal centred at 622 nm corresponding to the $Ru(bpy)_3^{2+}$ dye was observed on 250AT and DS850, having gold and carbon as working electrodes and Pt as a counter electrode in both cases. Other carbon (C110) or gold (220BT, 220AT) electrodes containing carbon or gold as counter electrodes offered lower signal, concluding that Pt as counter electrode also plays an important role for the ECL response. Finally, the ECL intensities obtained with transparent electrodes were much lower in comparison with the intensities found for the ceramic electrodes, which is presumably due to the thickness of the working electrodes employed. The ECL signal intensities increased on the order of P10 < AUTR10 ≤ 220AT < COTE10 ≤ 220BT << C110 <<< DS850 ≤ 250AT. Therefore, the SPE model 250AT, consisting of a working electrode of gold, a counter electrode of platinum and a reference electrode of silver, was chosen for all further experiments.

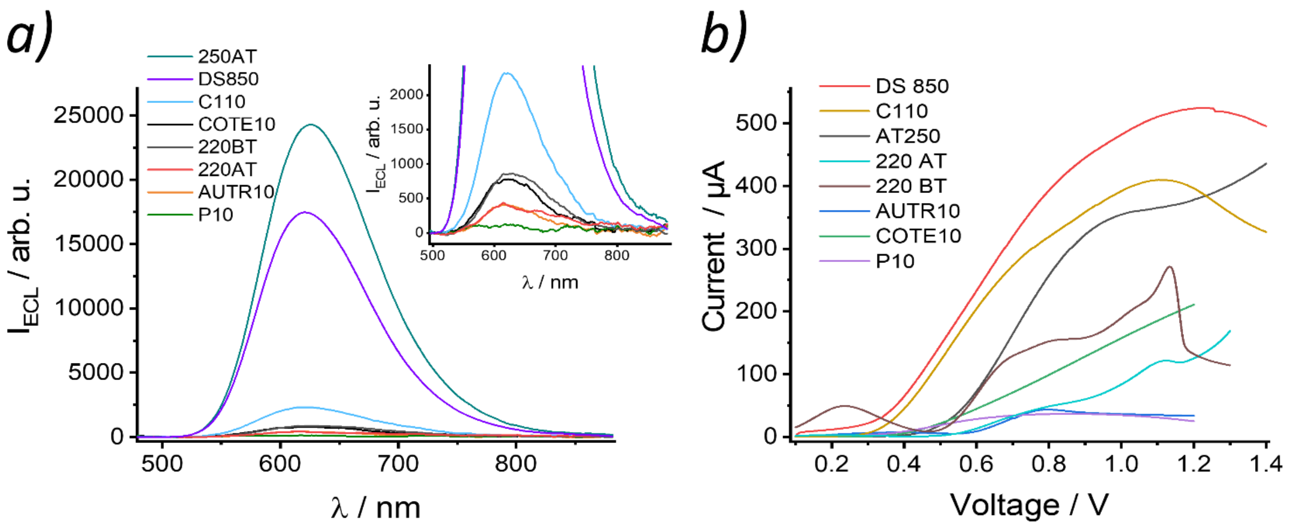

Figure S7. a) Maximum ECL observed under linear sweep voltammetry assay conditions as a function of the electrodes tested in presence of 10 µM of $Ru(bpy)_3^{2+}$ (PB 10 mM; NBEA 25 mM). b) Corresponding increase of the current as a function of the voltage applied.

5.3 Optimization of co-reactant concentration

After having selected the most suitable co-reactant and SPE electrode, optimization of the working concentration of co-reactant for maximum ECL generation is necessary, taking into account that a too high excess of co-reactant with respect to ECL reporter can lead to signal inhibition because of ECL self-quenching phenomena.[13] Such phenomena arise when side reactions between short-lived co-reactant intermediates and co-reactant start to dominate, decreasing the effective concentration of co-reactant intermediates and slowing down turnover through redox reactions at the electrode. The experiments were performed by spiking NBEA solutions between 0.8–25 mM into PB buffer (10 mM) containing $Ru(bpy)_3^{2+}$ at 0.65 µM. Amperometric measurements were performed on two different instruments, the spectraECL cell and a portable potentiostat device connected to a smartphone to register the light emitted with the help of the smartphone camera set in a 3D-printed black box to avoid the interference of ambient light (Figure S8). The intensity of the emitted light on the electrode was analyzed by extracting the integrated density of fluorescence of the red channel with the software ImageJ. As can be seen in Figure S8, both devices offered the same performance, and the optimum range of concentrations of NBEA was found to be between 3 and 6 mM.

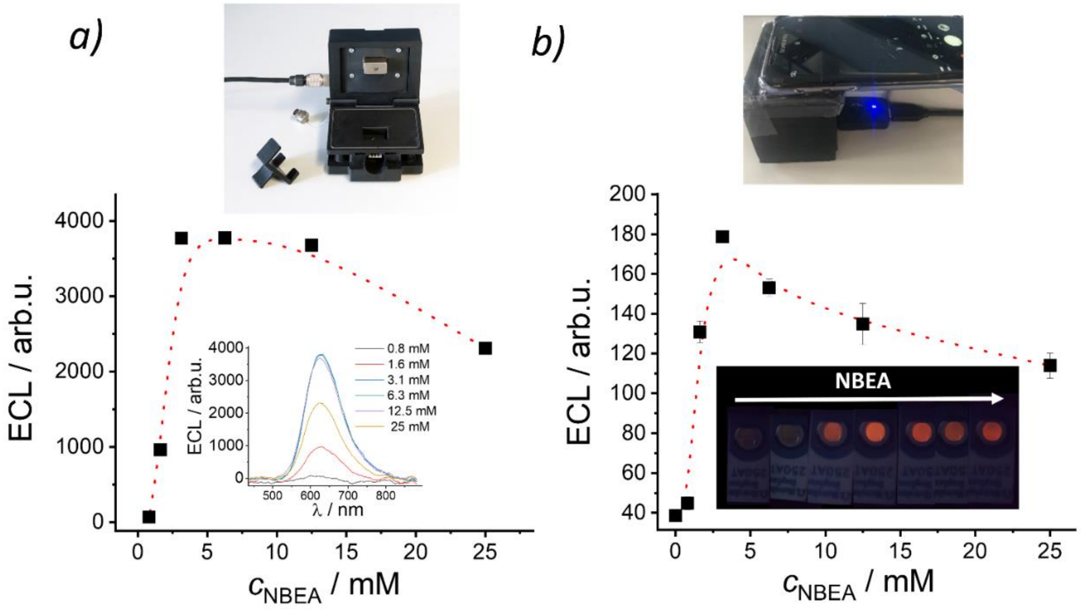

Figure S8. a) ECL signal registered at 620 nm of amperometry scans during 10 s of a solution of $Ru(bpy)_3^{2+}$ (0.65 µM) in presence of different amounts of NBEA performed with the spectraECL device (potential applied 1.2 V). Inset: Photograph of the spectraECL cell and corresponding emission spectra recorded as a function of the concentration of NBEA. b) Corresponding ECL signal recorded with the portable potentiostat connected to a smartphone. Insets: Photograph of the smartphone connected to the portable potentiostat via an OTG connection (top) and photograph of light emitted from the electrode as a function of the amount of NBEA employed, corresponding to the data points in the graph.

5.4 pH dependence of $Ru(bpy)_3^{2+}$ ECL emission

Working with buffered solutions yet a rather high amount of basic co-reactant, the influence of pH on the ECL emission yield of $Ru(bpy)_3^{2+}$ was assessed for choice of optimal assay parameters. Solutions of co-reactant NBEA (25 mM) in phosphate buffer (PB; 10 mM, pH 8) were thus spiked with $Ru(bpy)_3^{2+}$ to a final concentration of $c_{Ru(bpy)3}$ = 20 µM and the pH of the solution was adjusted from pH 6.0–10.5 with either HCl or NaOH. After pH adjustment, 50 µl of the solutions were deposited in the ECL cell of the spectraECL device and ECL emission was registered through LSV scans employing the SPE electrode C110 for the measurements in a similar way as described before. Figure S9 reveals that the ECL emission of $Ru(bpy)_3^{2+}$ increased up to pH 9, before decreasing again at higher pH.

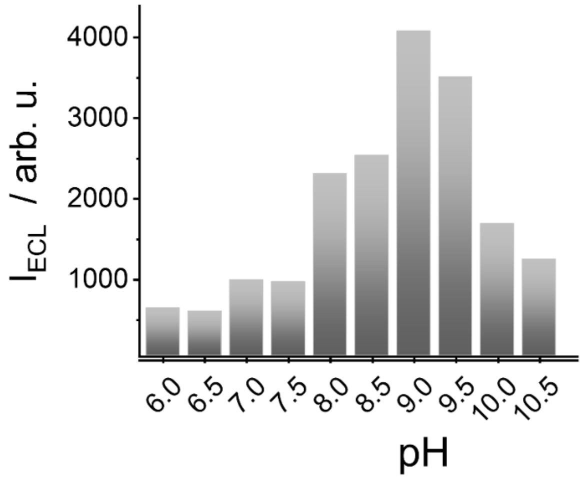

Figure S9. a) Maximum ECL observed under linear sweep voltammetry assay conditions at 1.2 V as a function of the pH tested in presence of 20 µM of $Ru(bpy)_3^{2+}$ (PB 10 mM; NBEA 25 mM).

5.5 Optimisation of co-reactant concentration in suspension assay

In a fist experiment, **MMRAA** was tested by suspending the material 5 min in the presence and the absence of 1 ppm of penicillin in a mixture of buffer containing different amounts of co-reactant and milk. Two aliquots of 10 µl of **MMRAA** (2 mg ml^{-1}) in PB (10 mM, pH 8) were added to 200 µl of a solution containing 50 µl of cow milk and 150 µl of NBEA at different concentrations (0.8–25 mM) in PB. One of the twin solutions was spiked with 2 µl of 100 ppm of penicillin, whereas to the other one was spiked with 2 µl of MilliQ water. The content of milk in the final solution is thus 25%. The pH remained constantly at pH 8.1 ± 0.1 for concentrations of NBEA lower than 6.25 mM, while it increased gradually to 8.7 and 9.1 for c_{NBEA} of 12.5 and 25 mM, respectively. The suspensions were stirred for 5 min, centrifuged and the ECL of $Ru(bpy)_3^{2+}$ released into the supernatant was measured with the smartphone camera connected to the portable Sensismart potentiostat through amperometry after applying a potential of 1.1 V for 10 s to the solutions. Results are shown in Figure 1 on the manuscript.

For the study of the response kinetics in suspension assays, the effectiveness of pore closure in the absence of penicillin was evaluated by dividing 100 µl of a **MMRAA** suspension (2 mg ml^{-1}) into two aliquots of 50 µl before dilution of both fractions with 250 µl of the co-reactant containing buffer (10 mM PB, pH 8; 5 mM NBEA) and 100 µl cow milk. One fraction was spiked with 4 µl of a penicillin stock solution (100 ppm), equivalent to a final analyte concentration of 1 ppm, whereas 4 µl water were added to the other fraction for the control sample. Fractions of both suspensions (70 µl) were collected after different time intervals, centrifuged (10,000 rpm, 1 min) and the ECL was measured amperometrically by applying a constant potential of 1.1 V for 10 s. The results are shown in Figure S10. The kinetics were also assessed for different amounts of penicillin at final concentrations between 1 ppb and 10 ppm, showing that the response accelerates with analyte concentration which hints at diffusion control of such assays in suspension (Figure S10b).

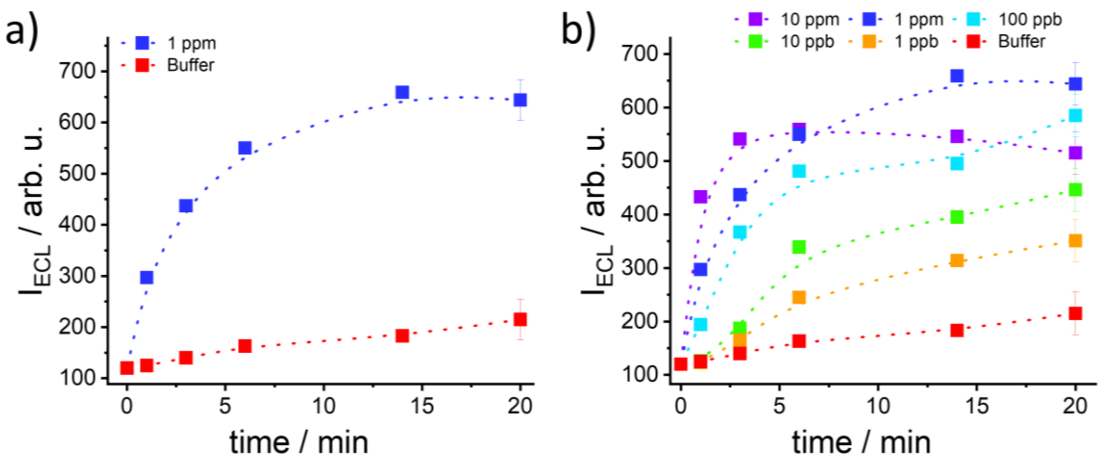

Figure S10 a) Increase in ECL of $Ru(bpy)_3^{2+}$ released from **MMRAA** vs time (PB 10 mM, pH 8; NBEA 5 mM) in the absence (red squares) and the presence (blue squares) of 1 ppm of penicillin. b) Increase in ECL of $Ru(bpy)_3^{2+}$ released from **MMRAA** vs time (PB 10 mM, pH 8; NBEA 5 mM) in the absence (red squares) and the presence of 10 ppm (purple squares), 1 ppm (blue squares), 100 ppb (cyan squares), 10 ppb (green squares) and 1 ppb (orange squares). The lines are included only as a guide to the eye for better illustration.

Following a similar procedure, system sensitivities were assessed by recoding dye release from **MMRAA** as a function of the concentration of penicillin after 5 min of reaction (Figure 2 in the main paper). The assays were performed by adding increasing amounts of penicillin to suspensions of 150 µl of **MMRAA** in buffer (10 mM PB, pH 8; 5 mM NBEA) containing 25% cow milk and stirring for 5 min after addition. After centrifugation, the ECL of the fraction of $Ru(bpy)_3^{2+}$ dyes that had been released into the supernatant was measured with the spectraECL device and with the smartphone. For comparison, the amount of $Ru(bpy)_3^{2+}$ released was also fluorometrically assessed (λ_{exc} 450 nm). The results are shown in Fig. S11.

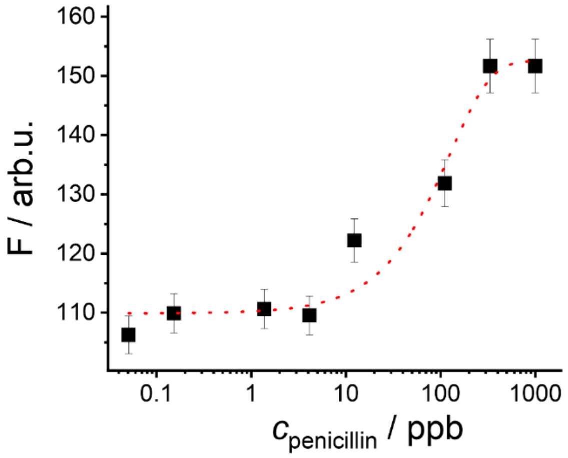

Figure S11. Fluorescence registered at 624 nm (λ_{ex} 450 nm) of the Ru(bpy) $_3^{2+}$ dye released from **MMRAA** as a function of the concentration of penicillin in milk (25%) diluted with buffer (PB 10 mM; NBEA 5 mM; 75%; pH 8) after 5 min of reaction. The lines exemplify four-parametric logistic fits.

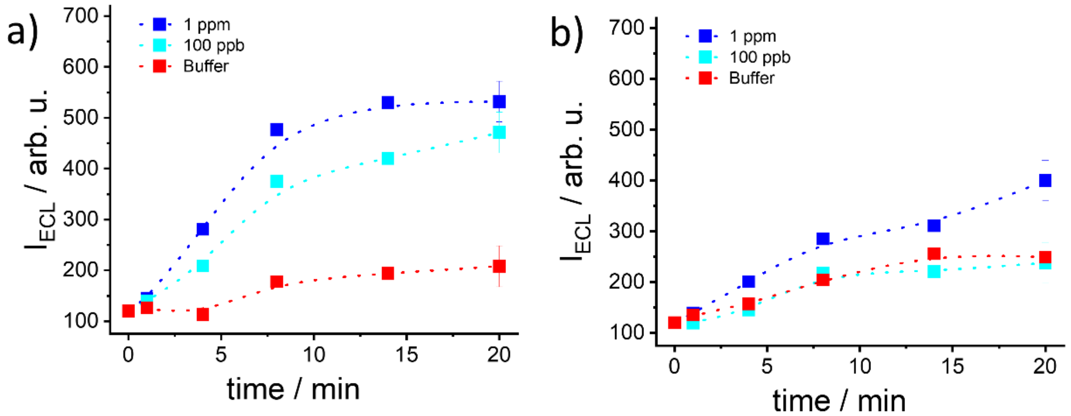

Figure S12. Increase in ECL of Ru(bpy) $_3^{2+}$ released from a) **MMRAAc** and b) **MMRCA** vs time (PB 10 mM, pH 8; NBEA 5 mM) in the absence (red squares) and the presence of 1 ppm (blue squares) or 100 ppb (cyan squares) of penicillinThe lines are included only as a guide to the eye for better illustration.

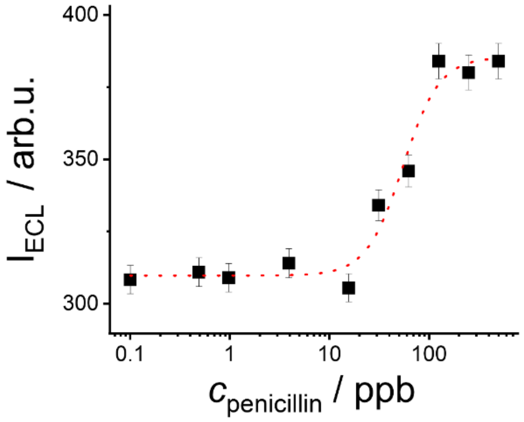

Figure S13. ECL signal of Ru(bpy) $_3^{2+}$ released from **MMRAAc** as a function of the concentration of penicillin in buffered milk (PB, 10 mM, pH 8; NBEA, 5 mM; 25% cow milk) after 5 min of reaction measured by the spectraECL cell.

6 Synthesis and characterization of paper strips

6.1 Synthesis of immersion-coated membranes ***(NP)GF***

In a falcon tube of 50 ml, 16 sheets of Fusion 5 membranes (10 x 2.5 cm) were immersed in 30 ml of a solution mixture of PB (10 mM) and *N*-butyldiethanolamine (NBEA; 50 mM) containing 0.1% of poly(ethyleneglycol). Membranes were stirred orbitally during 20 min at 120 rpm. Thereafter, the membranes were rinsed with phosphate buffer and dried in an oven at 85 °C for 12 h, yielding **(NP)GF**.

6.2 Synthesis of silane-functionalized membranes ***NPGF***

In a falcon tube of 50 ml, 16 sheets of Fusion 5 membranes (10 x 2.5 cm) were immersed in a mixture of toluene (28 ml), *N*-(3-triethoxysilylpropyl)diethanolamine (NPEAS; 1600 µl) and 3-[methoxy(polyethyleneoxy)propyl)] trimethoxysilane (PEGS; 800 µl). The membranes were stirred in a carrousel for 24 h at 40 rpm. Thereafter, the membranes were rinsed with EtOH and immersed for 15 min in EtOH, to remove adsorbed reagents from the membranes, before drying in an oven at 85 °C for 12 h.

In a second step, repetitive wax patterns of approximately 2.5 × 1 cm (the patterns guiding the flow and containing a circular detection zone of 8 mm in diameter at the end, to fit on the surface of the SPE electrode) was printed on an aluminium foil using a commercially available wax printer (Xerox Color Qube 8580; Figure S14). Next, the wax pattern was transferred to the **NPGF** membranes by lamination, before curing the strips at 110 °C for 1 h. This procedure melts the wax and creates hydrophobic barriers across the thickness of the glass fibre paper. The as-prepared **NPGF** were cut into individual strips of 2.5 × 1 cm before storing them at rt. Proper curing of the wax was tested by depositing 5 µl of a $Ru(bpy)_3^{2+}$ solution (1 mM) on the strips and inspecting it fluorometrically. Curing was considered successful when no leaching was observed outside of the hydrophobic wax patterns (Figure S14b).

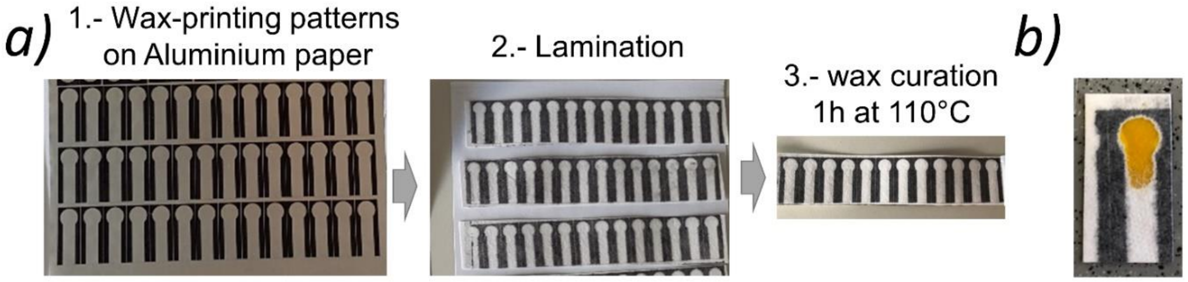

Figure S14. a) Procedure for the incorporation of hydrophobic patterns on **NPGF** membranes. b) Photograph of a **NPGF** strip containing the wax pattern after application of 5 µl of a $Ru(bpy)_3^{2+}$ solution (1 mM)

6.3 Incorporation of material ***MMRAA*** *into membrane* ***NPGF (MMRAA@NPGF)***

The sensing material **MMRAA** was incorporated into **NPGF** membranes by depositing 5 µl of a solution of **MMRAA** (2 mg mL^{-1}) in PB (10 mM) at the deposition zone A of the strip, located ca. 5 mm from the bottom end of the strip. The membranes were left to dry for 30 min at rt, before storing them at 8 °C in the refrigerator. Measurements were still reproducible after 3 months of storage. Thereafter, a decrease in the speed of the flow was observed, attributed to the hydrophobic wax barriers becoming slightly brittle.

6.4 Characterization of papers

The different modified papers were analyzed under an optical microscope and in more detail with an SEM. Images taken with the optical microscope showed that the incorporation of PEGS and NPEAS groups and also the subsequent incorporation of hydrophobic wax barriers lead to an expansion of the fibers, increasing the thickness of the strips from 335 ± 10 µm for **GF** to 465 ± 15 µm for **NPGF** (Figure S15a). Optical microscopy (Figure S15b) and SEM micrographs (Figure S15c) also showed that the step from **GF** to **NPGF** increases the roughness of the strips' surface. Finally, the presence of **MMRAA** particles on the papers was leading to an agglomeration of the particles around the fibers (see Figure S15c, image ii).

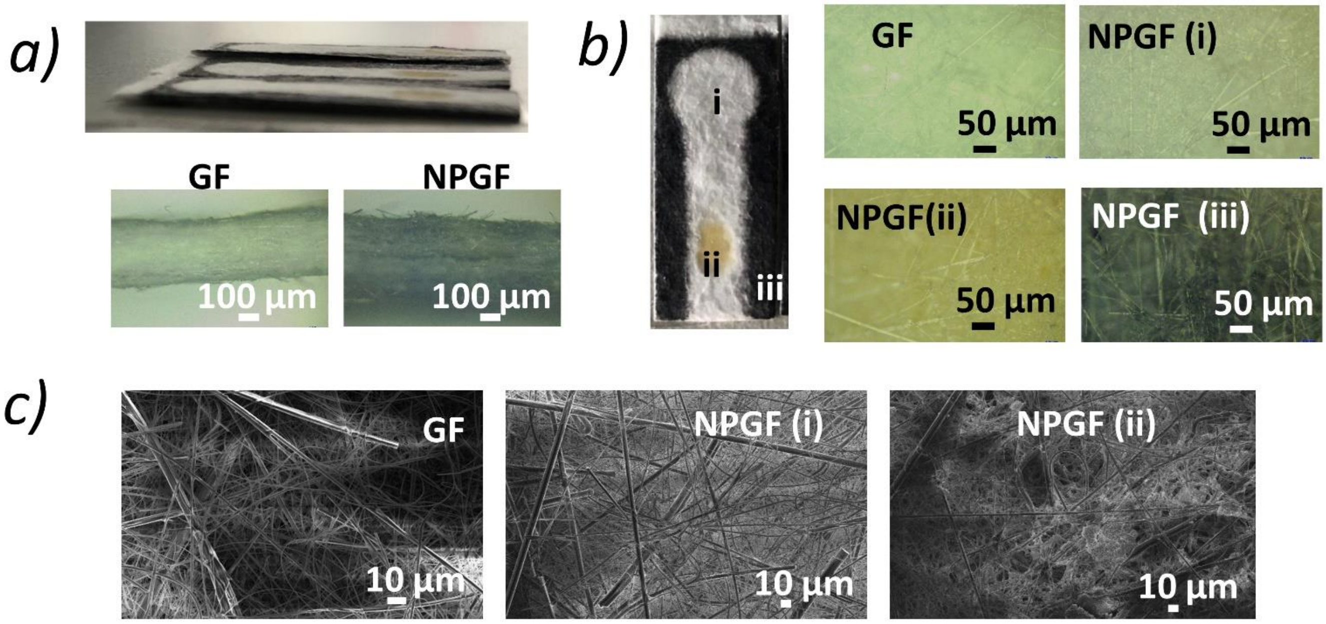

Figure S15. a) Images of the thickness of the fibres of **GF** and **NPGF** taken under an optical microscope. b) Optical microscope images of the fibres **GF** and **NPGF**: functionalised white zone (i), zone containing the sensing material **MMRAA** (ii) and black zone with hydrophobic wax barriers (iii). c) Representative SEM micrographs of the fibres of **GF** and **NPGF** in zones (i) and (ii).

TGA, EA and EDX analysis were also performed to qualitatively estimate the amounts of PEGS and NPEAS groups on the modified **NPGF** fibres. Figure S14a and Table S4 shows the mass loss determined from TGA analysis and also the average amount of C, N, Ru (in mmol g^{-1} SiO_2) for the reference membrane **GF** and for the modified paper **NPGF** in the functionalised white zone (i) and the zone containing the sensing material **MMRAA** (ii). Figure S16a shows the mass loss as a function of the temperature for the membranes **GF, NPGF** (i) and the wax part of **NPGF** (iii). TGA analysis revealed an increased mass loss of 6.7% for **NPGF** (i) with respect to **GF** membranes, having a total mass loss of 12.1%, ascribed to the PEG and NPEA moieties grafted on the paper. Furthermore, the incorporation of hydrophobic wax barriers produced an additional loss of mass of 9.6% (Table S4), amounting to a total mass loss of 21.7%.

EDX analysis also confirmed the presence of organic moieties with an increase in N and C for **NPGF**, whereas the presence of **MMRAA** on the paper could also be derived from a slight increase of the Ru, P, and S content, because of the presence of $Ru(bpy)_3^{2+}$ and the aptamer (Figure S16b and Table S4).

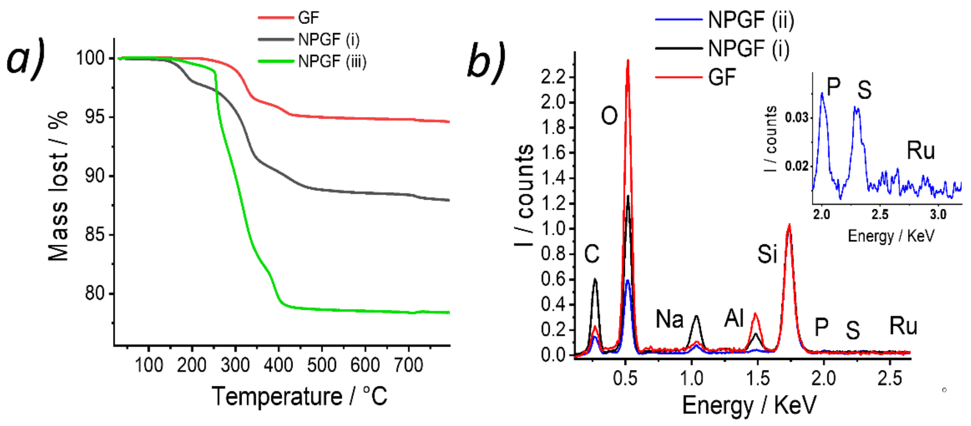

Figure S16. Mass loss (in %) of the papers **GF** and **NPGF** (i) and the wax part of **NPGF** (iii) as a function of the temperature as determined through TGA measurements. b) EDX intensity (normalised to Si) of the papers **GF**, **NPGF** (i) and **NPGF** containing **MMRAA** (ii).

Table S4. Mass lost (in %) estimated through TGA measurements and amounts of C, N and Ru (in mmol g SiO_2^{-1}) estimated through EDX analysis.

	Mass loss [%]	mmol C g^{-1} SiO_2[a]	mmol N g^{-1} SiO_2[a]	mmol P g^{-1} SiO_2[a]	mmol S g^{-1} SiO_2[a]	mmol Ru g^{-1} SiO_2[a]
**GF**	5.4	9.1 ± 3.9	2.5 ± 1.1	0.04 ± 0.02	0.09 ± 0.07	0.02 ± 0.01
**NPGF (i)**	12.1	35.8 ± 3.5	2.4 ± 0.3	0.05 ± 0.02	0.01 ± 0.03	0.02 ± 0.01-
**NPGF (ii)**	n.d.	12.0 ± 4.2	2.9± 0.5	0.45 ± 0.12	0.22 ± 0.07	0.10 ± 0.05
**NPGF (iii)**	21.7	n.d.	n.d.	n.d.	n.d.	n.d.

[a] The quantification obtained through SEM-EDX of the elemental composition is a very rough estimation due to the light elements C and N analysed. Due to this, data obtained can only be considered to compare the results obtained between the different papers analyzed.
n.d.: not determined

Finally, EA analysis resulted in 0.14 ± 0.02 and 0.09 ± 0.01 mmol g^{-1} solid of PEGS and NPEAS groups on the glass fibre membranes, corresponding to a total mass loss of 7.1 %, which is in good agreement with the data obtained by TGA.

7 Smartphone setup as readout device for fluorescence detection

The fluorescence of the $Ru(bpy)_3^{2+}$ dye released from **MMRAA** on the strips was measured with a smartphone camera, the smartphone being equipped with a 3D-printed case fabricated in an analogous way as reported previously by us.[15] The 3D-printed smartphone case contained an LED of 465 nm as excitation source powered by the smartphone via a USB-OTG link and filtered by a short-pass filter (532 nm) and a long-pass filter (550 nm) to collect the fluorescence emission after inserting the strip in the 3D-printed and customized holder.

8 Optimisation of strip-based assays

The improvement of the ECL efficiency of the modified papers was assessed by suspending 2.5 µl $Ru(bpy)_3^{2+}$ solution (1.2 mM) at ca. 7 mm from the bottom and dipping the strips for 2 min into 300 µl of a solution of PB 10 mM (pH 8) or PB 10 mM containing NBEA (25 mM). Thereafter, the strips were placed on the surface of an AT250 SPE electrode, and the ECL emission of the released $Ru(bpy)_3^{2+}$ was recorded in zone B with the spectraECL cell and with a smartphone connected to a portable potentistat. Fluorescence measurements were also performed to compare the ECL results with signals obtained with a 3D-printed holder, including an LED and filters connected to a smartphone (see previous Section 7 and our work in [15]). The results are shown in Figure 3 of the main paper.

In further experiments, considering the fast flow observed during the optimization of the NBEA concentration, the optimum time for dipping and flow development before ECL measurement was assessed. Thus, strips **MMRAA@NPGF** were dipped into 300 µl of buffered solutions containing 25% of milk and 25 mM NBEA co-reactant in presence and in absence of 1 ppm of penicillin (or none, for blank referencing), like described before, and left for the flow to develop for 1, 2, 5 or 10 min. Thereafter, the ECL of the dye released and collected in zone B was evaluated as detailed before. The optimum contrast between dye release in the presence and the absence of penicillin was found for a dipping time of 2 min (Figure S17), which is favourably fast for a rapid test and was accordingly used in all further experiments.

In another series of experiments, like for **MMRAA** in suspension, the optimum amount of co-reactant had to be verified. This was done by dipping the strips **MMRAA@NPGF** into 300 µl of buffered solutions containing 25% of milk, different amounts of NBEA co-reactant (3.1–50 mM) and 1 ppm of penicillin (or none, for blank referencing), in analogy to described before. The pH of the resulting mixtures increased gradually from 8.1–9.5 when NBEA was used at concentrations between 3.1–50 mM. A first observation was that the flow developed fast, reaching the top of the strip already after 20 s. To ensure complete travel of the reporter, the strips were left for 2 min in contact with the solution, the dipping time that was found to provide optimum contrast (Figure S17). Thereafter, the strips were placed on an AT250 SPE electrode, and the ECL emission of the released $Ru(bpy)_3^{2+}$ was recorded in zone B with a smartphone connected to a portable potentistat as described above. Figure 4 on the manuscript shows the corresponding reddish orange ECL light emitted in the absence and the presence of penicillin as a function of the concentration of NBEA employed.

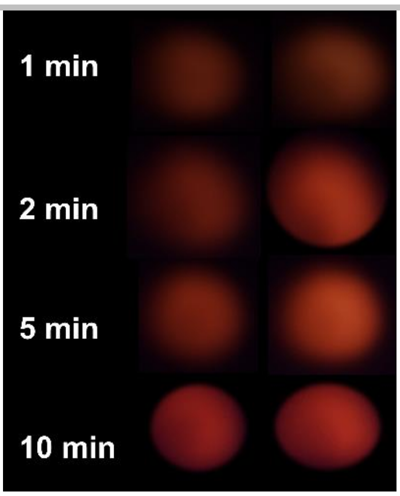

Figure S17. Photographs showing the ECL signal of $Ru(bpy)_3^{2+}$ being present in the detection zone B in the absence and the presence of 1 ppm of penicillin as a function of the time dipped (1, 2, 5 or 10 min) in the kit solution (PB 10 mM, pH 8, 25 mM NBEA) with which the sample (milk) is diluted in a ratio of 3+1 kit solution + sample. Photographs were registered during applying 1.1 V for 10 s in amperometric mode.

The sensitivity of the strips **MMRAA@NPGF** was evaluated next. The sensing membranes were dipped into 300 µl of buffered solutions (PB, 10 mM, pH 8; NBEA, 25 mM) containing 25% of milk and different amounts of penicillin as described before. ECL measurements were again performed with both setups. Furthermore, fluorescence measurements were also performed to compare the sensitivity of the traditional with the newly developed approach. The intensity of the fluorescence of the photographs of the strips was analysed in both cases by splitting the RGB channels of the images and extracting the integrated density of the red channel of fluorescence of the different zones B_i with the software ImageJ, following a similar approach as described above for ECL measurements with the portable potentiostat. The results are shown in Figure 5 of the main paper.

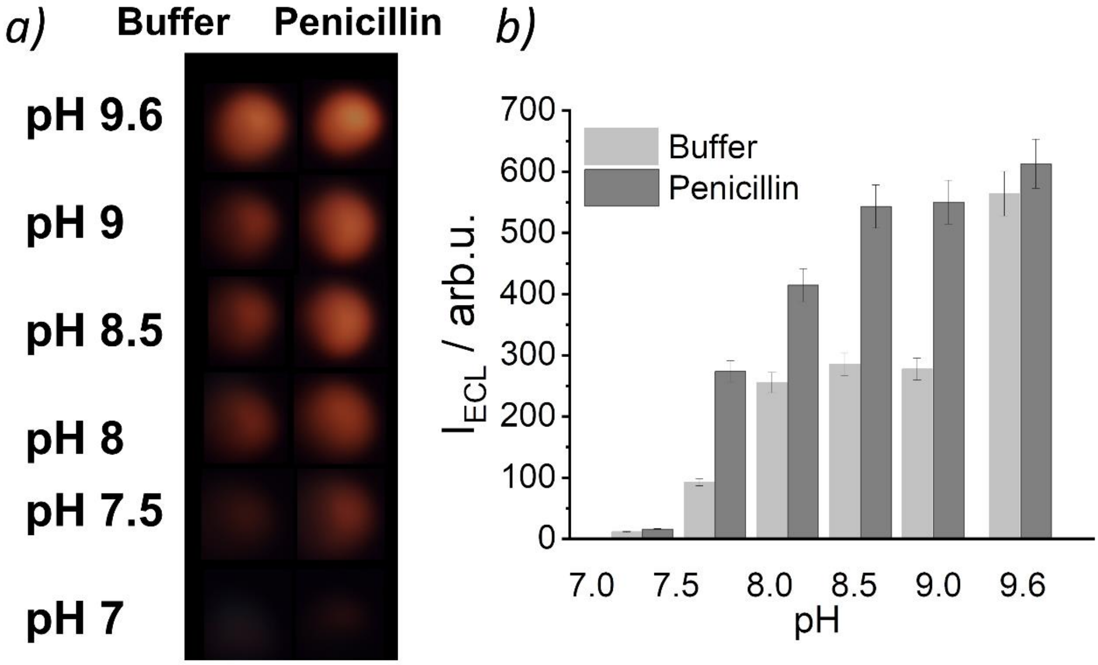

Figure S18. a) Photographs showing the ECL signal of $Ru(bpy)_3^{2+}$ being present in the detection zone B in the absence and the presence of 250 ppb of penicillin as a function of the pH (7-9.6) in the kit solution (PB 10 mM, pH 8, 25 mM NBEA) with which the sample (milk) is diluted in a ratio of 3+1 kit solution + sample. Photographs were registered during applying 1.1 V for 10 s in amperometry mode. b) Plot of the corresponding ECL signal as a function of the pH of the final solution in presence (dark grey) and in absence (grey) of 250 ppb of penicillin.

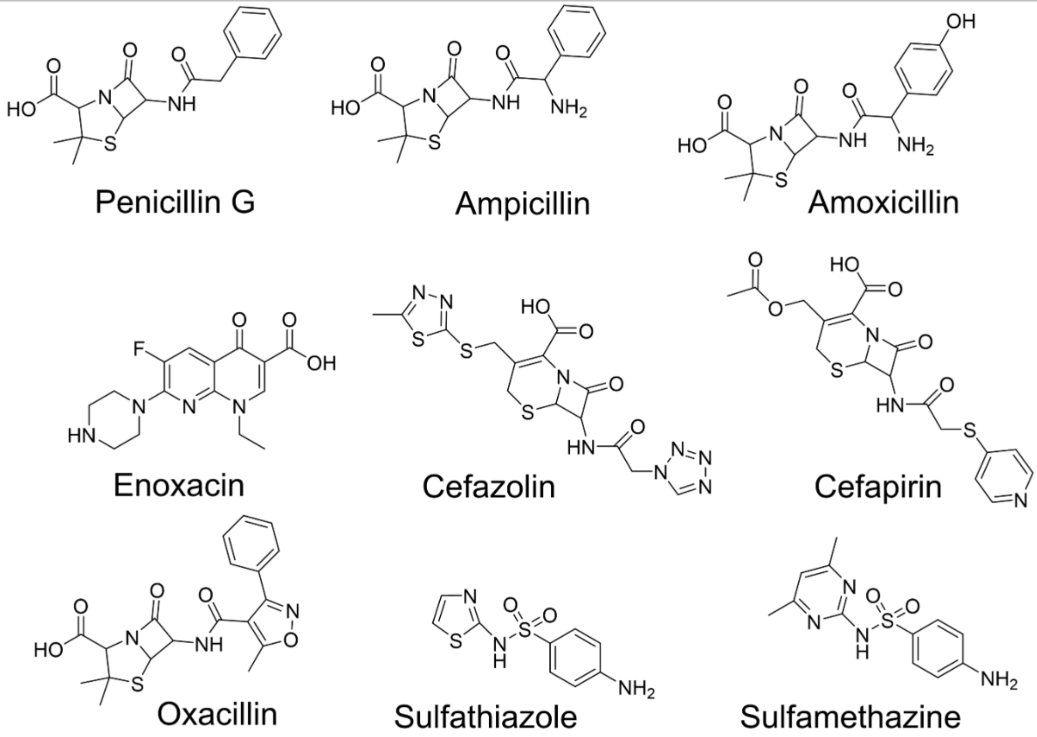

Figure S19. Chemical structure of antibiotics tested on **NPGF** membranes containing **MMRAA** sensing material.

9 Additional notes on analytical performance

9.1 Limits of detection

For the experiments in suspension, the ECL emission of the supernatants of the suspensions of the materials in the presence of different concentrations of penicillin were recorded, and the values of the fluorescence enhancement observed during the titrations were plotted vs. analyte concentration. These curves were fitted to a four-parameter logistic fitting function (equation S1).[16]

$$\frac{\boldsymbol{I}}{\boldsymbol{I}_0} = \frac{\boldsymbol{F}_1 - \boldsymbol{F}_2}{\left(1 + \frac{x}{x_0}\right)^P} + \boldsymbol{F}_2 \tag{S1}$$

Here, F_1 and F_2 correspond to the minimum and maximum enhancement of fluorescence observed, and p corresponds to the slope of the sigmoidal curve. Limits of detection (LODs), which describe the smallest concentration of analyte that can be reliably detected, were derived calculating in a first instance the Limits of Blank (LOBs; describing the smallest concentration of analyte that can be observed) and calculating the corresponding concentration of the signal as follows:[17]

LOB = mean blank + 1.645(σ_{blank})

LOD = LOB + 1.645($\sigma_{low\ concentration\ sample}$)

Generally, three repeat experiments were carried out for both the kinetics and the concentration-dependent measurements.

10 Author Contributions

E.C. and K.R. conceived the experiments. E.C. performed the experiments. E.C. and K.R. prepared the manuscript. Both authors discussed the results and commented on the manuscript.

References

[1] B. R. Pauw, A. J. Smith, T. Snow, N. J. Terrill, A. F. Thunemann, *J. Appl. Crystallogr.* **2017**, *50*, 1800-1811.
[2] V. Sreeja, K. N. Jayaprabha, P. A. Joy, *Appl. Nanosci.* **2015**, *5*, 435-441.
[3] D. Li, S. Egodawatte, D. I. Kaplan, S. C. Larsen, S. M. Serkiz, J. C. Seaman, *J. Hazard. Mater.* **2016**, *317*, 494-502.
[4] a) H. He, S.-Q. Wang, Z.-Y. Han, X.-H. Tian, W.-W. Zhang, C.-P. Li, M. Du, *Appl. Surf. Sci.* **2020**, *531*, 147342; b) J. Zhao, W. J. Guo, M. S. Pei, F. Ding, *Anal. Methods* **2016**, *8*, 4391-4397.
[5] E. Climent, R. Gotor, C. Tobias, J. Bell, P. M. Martin-Sanchez, K. Rurack, *ACS Sens.* **2021**, *6*, 27-34.
[6] M. Agthe, K. Høydalsvik, A. Mayence, P. Karvinen, M. Liebi, L. Bergström, K. Nygård, *Langmuir* **2015**, *31*, 12537-12543.
[7] X. Yu, Y. Zhu, *Sci. Technol. Adv. Mater.* **2016**, *17*, 229-238.
[8] D. Sarma, K. Gawlitza, K. Rurack, *Langmuir* **2016**, *32*, 3717-3727.
[9] a) S. Han, W. Niu, H. Li, L. Hu, Y. Yuan, G. Xu, *Talanta* **2010**, *81*, 44-47; b) S. Kirschbaum-Harriman, A. Duerkop, A. J. Baeumner, *Analyst* **2017**, *142*, 2648-2653.
[10] L. Xue, L. Guo, B. Qiu, Z. Lin, G. Chen, *Electrochem. Commun.* **2009**, *11*, 1579-1582.
[11] G. Valenti, A. Fiorani, H. Li, N. Sojic, F. Paolucci, *ChemElectroChem* **2016**, *3*, 1990-1997.
[12] J. Lee, D. W. M. Arrigan, D. S. Silvester, *Sens. Bio-Sens. Res.* **2016**, *9*, 38-44.
[13] L. Guo, L. Xue, B. Qiu, Z. Lin, D. Kim, G. Chen, *Phys. Chem. Chem. Phys.* **2010**, *12*, 12826-12832.
[14] a) Y. Hu, A. Cecconello, A. Idili, F. Ricci, I. Willner, *Angew. Chem. Int. Ed.* **2017**, *56*, 15210-15233; b) A. Porchetta, A. Idili, A. Vallée-Bélisle, F. Ricci, *Nano Lett.* **2015**, *15*, 4467-4471.
[15] E. Climent, M. Biyikal, D. Gröninger, M. G. Weller, R. Martínez-Máñez, K. Rurack, *Angew. Chem. Int. Ed.* **2020**, *59*, 23862-23869.
[16] Y. Xiang, J. Donley, E. Seletskaia, S. Shingare, J. Kamerud, B. Gorovits, *AAPS J.* **2018**, *20*, Art. No. 45.
[17] D. A. Armbruster, T. Pry, *Clin. Biochem. Rev. (Ultimo, Aust.)* **2008**, *29 Suppl 1*, S49-S52.
